# *PMP22* duplication dysregulates lipid homeostasis and plasma membrane organization in developing human Schwann cells

**DOI:** 10.1093/brain/awae158

**Published:** 2024-05-14

**Authors:** Robert Prior, Alessio Silva, Tim Vangansewinkel, Jakub Idkowiak, Arun Kumar Tharkeshwar, Tom P Hellings, Iliana Michailidou, Jeroen Vreijling, Maarten Loos, Bastijn Koopmans, Nina Vlek, Cedrick Agaser, Thomas B Kuipers, Christine Michiels, Elisabeth Rossaert, Stijn Verschoren, Wendy Vermeire, Vincent de Laat, Jonas Dehairs, Kristel Eggermont, Diede van den Biggelaar, Adekunle T Bademosi, Frederic A Meunier, Martin vandeVen, Philip Van Damme, Hailiang Mei, Johannes V Swinnen, Ivo Lambrichts, Frank Baas, Kees Fluiter, Esther Wolfs, Ludo Van Den Bosch

**Affiliations:** Department of Neurosciences, Experimental Neurology and Leuven Brain Institute (LBI), KU Leuven—University of Leuven, Leuven 3000, Belgium; Laboratory of Neurobiology, VIB, Center for Brain & Disease Research, Leuven 3000, Belgium; Department of Ophthalmology, Medical Faculty, University of Bonn, Bonn 53127, Germany; Department of Neurosciences, Experimental Neurology and Leuven Brain Institute (LBI), KU Leuven—University of Leuven, Leuven 3000, Belgium; Laboratory of Neurobiology, VIB, Center for Brain & Disease Research, Leuven 3000, Belgium; Laboratory of Neurobiology, VIB, Center for Brain & Disease Research, Leuven 3000, Belgium; UHasselt—Hasselt University, Biomedical Research Institute, Diepenbeek 3590, Belgium; Laboratory of Lipid Metabolism and Cancer, Department of Oncology, KU Leuven, Leuven 3000, Belgium; Department of Analytical Chemistry, Faculty of Chemical Technology, University of Pardubice, Pardubice 532 10, Czech Republic; Department of Neurosciences, Experimental Neurology and Leuven Brain Institute (LBI), KU Leuven—University of Leuven, Leuven 3000, Belgium; Laboratory of Neurobiology, VIB, Center for Brain & Disease Research, Leuven 3000, Belgium; Department of Clinical Genetics, Leiden University Medical Center, Leiden 2333 ZA, The Netherlands; Department of Clinical Genetics, Leiden University Medical Center, Leiden 2333 ZA, The Netherlands; Department of Clinical Genetics, Leiden University Medical Center, Leiden 2333 ZA, The Netherlands; InnoSer Nederland B.V., 2333 CK Leiden, The Netherlands; InnoSer Nederland B.V., 2333 CK Leiden, The Netherlands; InnoSer Nederland B.V., 2333 CK Leiden, The Netherlands; Department of Biomedical Data Sciences, Sequencing Analysis Support Core, Leiden University Medical Center, Leiden 2333 ZA, The Netherlands; Department of Biomedical Data Sciences, Sequencing Analysis Support Core, Leiden University Medical Center, Leiden 2333 ZA, The Netherlands; Department of Neurosciences, Experimental Neurology and Leuven Brain Institute (LBI), KU Leuven—University of Leuven, Leuven 3000, Belgium; Laboratory of Neurobiology, VIB, Center for Brain & Disease Research, Leuven 3000, Belgium; Department of Neurosciences, Experimental Neurology and Leuven Brain Institute (LBI), KU Leuven—University of Leuven, Leuven 3000, Belgium; Laboratory of Neurobiology, VIB, Center for Brain & Disease Research, Leuven 3000, Belgium; Department of Neurosciences, Experimental Neurology and Leuven Brain Institute (LBI), KU Leuven—University of Leuven, Leuven 3000, Belgium; Laboratory of Neurobiology, VIB, Center for Brain & Disease Research, Leuven 3000, Belgium; Department of Neurosciences, Experimental Neurology and Leuven Brain Institute (LBI), KU Leuven—University of Leuven, Leuven 3000, Belgium; Laboratory of Neurobiology, VIB, Center for Brain & Disease Research, Leuven 3000, Belgium; Laboratory of Lipid Metabolism and Cancer, Department of Oncology, KU Leuven, Leuven 3000, Belgium; Laboratory of Lipid Metabolism and Cancer, Department of Oncology, KU Leuven, Leuven 3000, Belgium; Department of Neurosciences, Experimental Neurology and Leuven Brain Institute (LBI), KU Leuven—University of Leuven, Leuven 3000, Belgium; Laboratory of Neurobiology, VIB, Center for Brain & Disease Research, Leuven 3000, Belgium; Department of Neurosciences, Experimental Neurology and Leuven Brain Institute (LBI), KU Leuven—University of Leuven, Leuven 3000, Belgium; Laboratory of Neurobiology, VIB, Center for Brain & Disease Research, Leuven 3000, Belgium; Clem Jones Centre for Ageing Dementia Research, Queensland Brain Institute, The University of Queensland, Brisbane, QLD 4072, Australia; Clem Jones Centre for Ageing Dementia Research, Queensland Brain Institute, The University of Queensland, Brisbane, QLD 4072, Australia; School of Biomedical Sciences, The University of Queensland, Brisbane, QLD 4072, Australia; UHasselt—Hasselt University, Biomedical Research Institute, Diepenbeek 3590, Belgium; Department of Neurosciences, Experimental Neurology and Leuven Brain Institute (LBI), KU Leuven—University of Leuven, Leuven 3000, Belgium; Laboratory of Neurobiology, VIB, Center for Brain & Disease Research, Leuven 3000, Belgium; Department of Neurology, University Hospitals Leuven, Leuven 3000, Belgium; Department of Biomedical Data Sciences, Sequencing Analysis Support Core, Leiden University Medical Center, Leiden 2333 ZA, The Netherlands; Laboratory of Lipid Metabolism and Cancer, Department of Oncology, KU Leuven, Leuven 3000, Belgium; UHasselt—Hasselt University, Biomedical Research Institute, Diepenbeek 3590, Belgium; Department of Clinical Genetics, Leiden University Medical Center, Leiden 2333 ZA, The Netherlands; Department of Clinical Genetics, Leiden University Medical Center, Leiden 2333 ZA, The Netherlands; UHasselt—Hasselt University, Biomedical Research Institute, Diepenbeek 3590, Belgium; Department of Neurosciences, Experimental Neurology and Leuven Brain Institute (LBI), KU Leuven—University of Leuven, Leuven 3000, Belgium; Laboratory of Neurobiology, VIB, Center for Brain & Disease Research, Leuven 3000, Belgium

**Keywords:** Charcot–Marie–Tooth disease type 1A, human induced pluripotent stem cells, Schwann cells, lipid metabolism, plasma membrane, lipid storage

## Abstract

Charcot–Marie–Tooth disease type 1A (CMT1A) is the most common inherited peripheral neuropathy caused by a 1.5 Mb tandem duplication of chromosome 17 harbouring the *PMP22* gene. This dose-dependent overexpression of *PMP22* results in disrupted Schwann cell myelination of peripheral nerves. To obtain better insights into the underlying pathogenic mechanisms in CMT1A, we investigated the role of *PMP22* duplication in cellular homeostasis in CMT1A mouse models and in patient-derived induced pluripotent stem cells differentiated into Schwann cell precursors (iPSC-SCPs).

We performed lipidomic profiling and bulk RNA sequencing (RNA-seq) on sciatic nerves of two developing CMT1A mouse models and on CMT1A patient-derived iPSC-SCPs. For the sciatic nerves of the CMT1A mice, cholesterol and lipid metabolism was downregulated in a dose-dependent manner throughout development. For the CMT1A iPSC-SCPs, transcriptional analysis unveiled a strong suppression of genes related to autophagy and lipid metabolism. Gene ontology enrichment analysis identified disturbances in pathways related to plasma membrane components and cell receptor signalling. Lipidomic analysis confirmed the severe dysregulation in plasma membrane lipids, particularly sphingolipids, in CMT1A iPSC-SCPs. Furthermore, we identified reduced lipid raft dynamics, disturbed plasma membrane fluidity and impaired cholesterol incorporation and storage, all of which could result from altered lipid storage homeostasis in the patient-derived CMT1A iPSC-SCPs. Importantly, this phenotype could be rescued by stimulating autophagy and lipolysis.

We conclude that *PMP22* duplication disturbs intracellular lipid storage and leads to a more disordered plasma membrane owing to an alteration in the lipid composition, which might ultimately lead to impaired axo-glial interactions. Moreover, targeting lipid handling and metabolism could hold promise for the treatment of patients with CMT1A.

## Teaser

Peripheral nerve insulation requires an intricate relationship between neurons and Schwann cells which is highly dependent on lipids. Prior *et al.* show how an excess of the PMP22 protein dysregulates the storage of lipids and their incorporation into the plasma membrane of Schwann cells, giving rise to Charcot–Marie–Tooth disease type 1A.

## Introduction

Duplications of the *PMP22* gene, which encodes peripheral myelin protein 22 (PMP22), are responsible for the most prevalent form of Charcot–Marie–Tooth disease (CMT), known as CMT1A.^1,2^ CMT1A patients exhibit a dysmyelinating phenotype in their peripheral nerves and may experience mild-to-severe muscular atrophy in the distal regions of their body, with some also presenting with sensory abnormalities.^3^ PMP22 is a membrane glycoprotein constituting 2%–5% of compact myelin of the peripheral nervous system, which is produced primarily by Schwann cells.^4^

Myelin is a complex and intricate structure that composes both compacted and non-compacted membranes and it is essential not only for the maturation of associated axons, but also for accelerating nerve conduction propagation.^5,6^ Myelin consists of ∼70%–80% lipids, which are mainly fatty aldehydes and very long-chain fatty acids. The remaining 20%–30% are proteins, and >60% of these are glycoproteins.^7-9^ Cholesterol, a critical component of the myelin membrane, exerts a significant influence on myelination through its impact on glial cell differentiation, myelin membrane biogenesis and the formation of functional myelin.^10^

In the plasma membrane (PM), cholesterol is key in the regulation of membrane fluidity. It can be partitioned into more ordered and stable phases than the surrounding fluid phase, where it is enriched together with a specific type of sphingolipid (SP), called sphingomyelin (SM). These liquid-ordered domains are called lipid rafts and are specialized regions of the PM that segregate proteins with different functions, including the shuttling of molecules across the cell surface and the organization of cell signal transduction pathways.^11-13^ Importantly, lipid rafts provide a platform for the clustering of multiple molecules, such as lipids and receptors, allowing for efficient and specific signalling during myelination. Here, cholesterol helps to maintain the fluidity and stability of the PM.^14,15^ Cholesterol trafficking and the establishment of lipid rafts on the cell membrane are impaired in *Pmp22*-deficient mice.^16,17^ However, it is unknown whether CMT1A patient cells have an altered lipid metabolism or impaired lipid rafts.

The storage of cholesterol and lipids in eukaryotic cells occurs within lipid droplets (LDs), functioning as an endogenous source of lipids for the synthesis of biomembranes. From here, fatty acids can be mobilized by the cell during nutrient stress, cell differentiation or biomembrane repair via lipophagy (i.e. lysosome-mediated LD recycling or breakdown) or enzyme-mediated lipolysis, which is executed primarily via adipose triglyceride lipase (ATGL) (reviewed by Olzmann and Carvalho^18^).^19,20^ During lipid starvation, cells initially produce LDs as a short-term response, followed by their breakdown and utilization when starvation persists (reviewed by Henne *et al.*^21^).

Until now, the pathogenic mechanisms underlying CMT1A aetiology were studied mainly using *PMP22*-overexpressing rodent models, such as the C22 and the C3 CMT1A transgenic models and the transgenic CMT1A rat model.^22,23^ These CMT1A rodent models overexpress different copy number levels of human *PMP22*, giving rise to an array of disease severities. Recently, the development of human induced pluripotent stem cells (iPSCs) has facilitated the study of patient-derived cells.

In this study, our aim was to investigate the effect of *PMP22* duplication in a physiological context using a human model and to compare it with sciatic nerves of CMT1A mouse models. Therefore, we used mice overexpressing *PMP22* and Schwann cell precursors derived from CMT1A patient iPSCs (iPSC-SCPs). We discovered that increased *PMP22* copy number dysregulated genes relating to myelination, autophagy and lipid metabolism at this early Schwann cell lineage stage, which resulted in altered lipid flux. This alteration in the lipidome was characterized by changes in lipid storage homeostasis and the PM lipid composition. Moreover, this resulted in disordered PM lipid properties and altered lipid raft dynamics. We were able to restore the cholesterol and lipid defects in CMT1A patient-derived iPSC-SCPs by specifically targeting lipid storage and the release of free cholesterol. Altogether, our study highlights the underlying pathogenic mechanisms of *PMP22* duplication by its regulation of lipid storage and of lipid trafficking to the PM, suggesting that these aspects of lipid metabolism are a potential target for therapy in CMT1A patients.

## Materials and methods

### Animals

Animal experiments were conducted either at Leiden University Medical Center (LUMC) or at KU Leuven.

All the experiments at LUMC were done after ethical approval by the institutional ethical committee and the central (national) commission for animal experiments according to EU directive 2010/63/EU. The C22-PMP22 mouse model (C22 mice) and its spontaneous revertant, the C3-PMP22 mouse model (C3 mice),^24^ were both bred heterozygous using wild-type (WT) males × heterozygous females and kept specific pathogen free at Janvier-labs (Le Genest Saint Isle, France). Animals were sent to the LUMC or the Free University (InnoSer Nederland B.V.) for experiments and allowed to acclimate for ≥1 week. All animals were housed socially with at least three mice per cage. The animal room was maintained at a temperature of 21°C ± 2°C, with a relative humidity between 45% and 65%, and a 12 h–12 h light–dark cycle. All cages were provided with sawdust as bedding material, and food (commercial rodent diet) and drinking water were provided *ad libitum*.

All procedures at KU Leuven were conducted in accordance with the ethical standards for experiments on animals established and approved by the Animal Ethics Committee of KU Leuven. Experiments were conducted under the ethical committee approval no. 104/2017. Breeding was done at KU Leuven, and the C3 mice were maintained in a C57BL6/J genetic background. Animals were kept and bred in the same conditions as described above.

To verify the genotype and copy number of human *PMP22*, we used droplet digital PCR (ddPCR) as reported previously^25^ and described further below.

### iPSC cultures

The CMT1A line, CS67iCMT-n1, and its isogenic control iPSC line, isogenic-CS67iCMT, were generated from fibroblasts isolated from a male CMT1A patient, as described before.^26^ These cell lines were produced by the Cedars-Sinai hiPSC core facility and are available at https://csbiomfg.com/cellcollection/. Confirmation of *PMP22* duplication was carried out using ddPCR as described below. iPSCs were maintained in Essential 8™ (E8) flex medium (Gibco) with penicillin/streptomycin (1000 U/ml) and cultured as described before.^27,28^ In brief, colonies were passaged each week with 0.5 mM EDTA (Invitrogen) diluted in Dulbecco’s phosphate-buffered saline (DPBS; Sigma-Aldrich) and plated on Matrigel LDEV-free, growth factor reduced (GFR) basement membrane matrix (Corning®). The absence of mycoplasma contamination was checked routinely by PCR.

### Droplet digital PCR

Droplet digital PCR was used to confirm *PMP22* duplication in the CMT1A line and its absence in the isogenic control. A qPCR assay probe (6-FAM/ZEN/IBFQ) was designed using the Integrated DNA Technology (IDT) RealTime PCR primer design tool based on the location at which the *PMP22* duplication in the isogenic iPSC line was corrected, as documented before.^26^ The following human *PMP22* primers and probes were used: forward, 5′-AGGCAGAAACTCCGCTG-3′; reverse, 5′-ACGAACAGCAGCACCAG-3′; and probe, 5′-CGATGATACTCAGCAACAGGAGGCA-3′.

The reference gene used in combination with the designed human *PMP22* assay was the commercially established *AP3B1* (dHsaCP1000001; #10031245, Bio-Rad) ddPCR copy number variation assay.

### Differentiation of Schwann cell precursors from human iPSCs

The protocol to generate iPSC-SCPs was adapted from Kim *et al*.^29^ Briefly, iPSCs were split into small colonies or a single-cell state and seeded on Matrigel-GFR in E8 flex medium 24 h before initiation of the protocol. RevitaCell™ supplement (Thermo Fisher Scientific; #A2644501) was added at 10 µM when seeding the cells. iPSC medium was then switched to neural differentiation medium, which was composed of a 1:1 mix of advanced DMEM/F12, GlutaMAX™ Supplement (Thermo Fisher Scientific, #10565018) and Neurobasal™ medium (Thermo Fisher Scientific, #21103049), containing 0.005% bovine serum albumin (BSA; Sigma-Aldrich, #A7979), B27 without vitamin A (1×; Thermo Fisher Scientific, #12587010), N2 (1×; Life Technology), 0.11 mM β-mercaptoethanol (Thermo Fisher Scientific), 2 mM GlutaMAX™ (Thermo Fisher Scientific), 3 µM CHIR-99021 (Tocris Biosciences) and 20 µM SB 431542 (Tocris Biosciences). After 6 days, the neural differentiation medium was supplemented with 50 ng/ml neuregulin1-β1 (NRG1) (Peprotech, #100-03), which is henceforth denoted as iPSC-SCP differentiation medium (SCPDM).

### Differentiation of Schwann cell precursors to Schwann cell-like cells

On Day 27 of the protocol, cells were seeded on poly-L-ornithine (100 μg/ml) and laminin (5 μg/ml) coated plates in SCPDM. The following day, the medium was switched to iPSC-SC induction medium, which is: DMEM-F12 supplemented with 100 U/ml penicillin/streptomycin, 40 µM SB 431542, 6 µM Chir 99021, 200 nM retinoic acid (Sigma-Aldrich, #R2625), 20 ng/ml platelet-derived growth factor-BB (PDGF-bb) (Peprotech, #100-14B), 5 µM forskolin (FSK) (STEMCELL Technologies, #100-0249) and 200 ng/ml NRG1. On Day 30, the medium was changed to the following composition: DMEM-F12 supplemented with 100 U/ml penicillin/streptomycin, 0.005% BSA, 20 µM SB 431542, 3 µM Chir 99021, 100 nM retinoic acid, 10 ng/ml PDGF-bb, 5 µM FSK and 200 ng/ml NRG1. On Day 32, the medium was switched, and cells were maintained in the final medium composition: DMEM-F12 supplemented with 100 U/ml penicillin/streptomycin, 0.005% BSA, 5 µM FSK and 200 ng of NRG1. iPSC-SCs were assessed at Day 35. However, iPSC-SCs can be maintained in culture for several weeks.

### Quantitative PCR and transcriptomics

RNA was purified from cell lysates as mentioned above via TRIzol™ reagent. The SuperScript™ III First-Strand Synthesis System (Invitrogen) was used to convert RNA (500 ng) to cDNA, according to the manufacturer’s instructions. For qPCR, Fast SYBR™ Green Master Mix (Applied Biosystems) was used, and the run was performed with the StepOne™ Real-Time PCR System (Applied Biosystems). The genes of interest obtained from the data were normalized to the reference genes *GAPDH* and *ACTB* and analysed further with qbase+ (Biogazelle). An overview of the primers used in this study is given in Supplementary Table 1.

### Immunocytochemistry

For membrane protein staining, cells were fixed with 4% paraformaldehyde for 20 min at room temperature and washed (three times) in Dulbecco's phosphate-buffered saline (DPBS +/+, Gibco Invitrogen). For structural proteins, cells were fixed with ice-cold methanol at −80°C for 10 min, followed again by washing (three times) with DPBS +/+. Cells were then blocked and permeabilized using 5% normal donkey serum (Sigma-Aldrich), 5% goat serum (Sigma-Aldrich) and 0.1% Triton X-100 (Acros Organics™) in PBS for 30 min. Primary antibodies were diluted 1:50–1:1000 in a blocking buffer (2% normal donkey serum in PBS) overnight at 4°C (Supplementary Table 2). The following day, after washing (three times) with DPBS +/+, incubation with the appropriate secondary antibodies labelled with Alexa Fluor 488, 555 or 647 (Invitrogen) at 1:1000 dilution was performed for 2 h at room temperature. Cells were incubated with NucBlue® (Thermo Fisher Scientific, #R37606) or NucRed® (Thermo Fisher Scientific, #R37106) in DPBS for 20 min, washed (three times), and mounted on glass slides using ProLong® Gold antifade reagent (Invitrogen, #P36934). Images were acquired using a DMi8 (Leica) confocal microscope. Images were analysed using the widely used non-commercial software ImageJ FIJI (NIH, Bethesda, MD, USA).

### Western blot analysis

At Day 0, iPSC-SCPs were seeded in SCPDM, and the following morning the medium was switched to a BSA-free SCPDM. Time point 0 h was collected at this moment; thereafter, cells were collected every 24 h for a total of 120 h. For protein collection, cells were washed with ice-cold DPBS and immediately lysed in RIPA buffer (Sigma-Aldrich) supplemented with cOmplete™ Mini, EDTA-free protease inhibitor cocktail (Roche) according to the manufacturer’s instructions. Determination of protein concentration, loading of the western blot and subsequent analysis were done as previously described.^25,30^ Each marker investigated was normalized to the total protein stain. All blots used for the quantifications are shown in Supplementary Fig. 1 with their corresponding total protein-stained blots that were used for normalization.

### Additional methods

Detailed descriptions of following methods are provided in the Supplementary material: sciatic nerve immunohistochemistry; transmission electron microscopy on iPSCs; *in vivo* and *in vitro* RNA sequencing and pathway analysis; lipidomic sample preparation, procedure and the notation for lipid structures; analysis of membrane polarity; lipid raft dynamic assay and analysis; high-throughput image analysis; and the myelination assay.

### Statistics

Investigators were blinded to the experimental conditions, and automated software programs were used, which guarantee objectivity of the analyses. Most statistical analyses were performed using GraphPad Prism software v.9 (GraphPad Software Inc., CA, USA). All data were first checked for normality to select the appropriate statistical test. Unless stated otherwise, Student’s unpaired two-tailed *t*-test was used for the comparison of two means and a one-way ANOVA was performed for the multiple-group analysis.

For giant plasma membrane vesicle (GPMV) phasor plots, programming in R (https://www.r-project.org/; v.4.2.2) was done to perform multivariate ANOVA to obtain data on group distribution (isogenic versus CMT1A) and η^2^. For raft dynamics, the Welch-corrected two-tailed *t*-test was used to compare the area under the curves (AUCs) and the mobile:immobile fractions. Statistical analysis for the lipidomics data was performed using the MetaboAnalyst platform (v.5.0),^31,32^ R programming language for statistical computing and graphics (v.4.2.2) and Cytoscape software (v.3.9.1). Graphics were merged using the Inkscape open-source vector graphics editor (v.1.2). Before performing statistical computations, all matrices were filtered, and lipids with >60% missing concentrations were removed. Next, the missing values were substituted for each lipid separately by 80% of the lowest concentration measured. Means with standard deviations (SD) were computed for each group using rstatix library (v.0.7.1). The Welch *t*-test was used for comparing the mean between CMT1A versus isogenic cells. Fold change was computed as a ratio of the mean value in CMT1A to the mean value in isogenic (cells) or WT (mice). Bubble plots reflecting simplified lipid metabolism were generated with the Cytoscape software. In both bubble plots, raw *P*-values from the Welch *t*-test were presented as −log_10_ and fold change values as log_2_. Volcano plots were prepared using EnhancedVolcano library in R (v.1.16.0) with the cut-off points of 0.3 for the log_2_ fold change (CMT1A to isogenic) and 0.05 raw *P*-value from the Welch *t*-test (rstatix library). Principal component analysis (PCA) plots were designed using the MetaboAnalyst v.5.0 platform after the log_10_-transformation of concentrations and Pareto scaling. Data are presented either as the mean ± standard error of the mean (SEM) or SD, as specified in the figure legends. Statistical significance was set as follows: *P < 0.05, **P < 0.01, ***P < 0.001 and ****P < 0.0001. All figures were assembled and finalized in Adobe Illustrator (v.2022).

### Ethics approval and consent to participate

The human iPSC line was obtained from a CMT1A patient with consent.

## Results

### Cholesterol biosynthesis is the major dysregulated pathway in CMT1A mice

**Figure 1:**
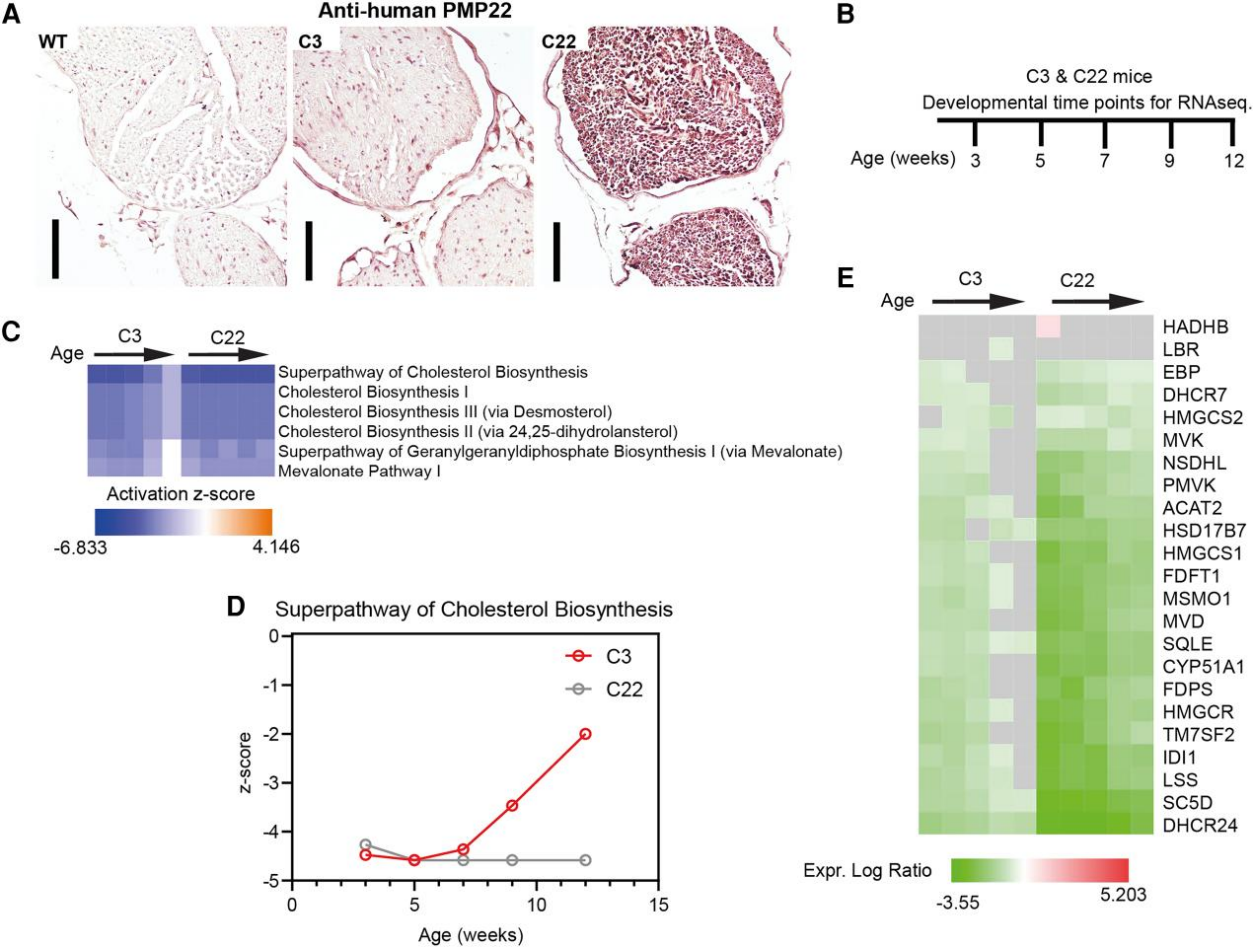
**Cholesterol biosynthesis is the major dysregulated pathway during nerve development of CMT1A mice.** (**A**) Immunohistochemical analysis of human PMP22 in the sciatic nerves of the C3 and C22 mouse models at 12 weeks of age. (**B**) Schematic overview of the time points analysed for bulk RNA sequencing of sciatic nerves isolated from the C3 and C22 mouse models, with their age-matched littermate controls used for normalization. (**C**) Activation heat map of the main canonical pathways (the cholesterol biosynthesis-related pathways) that are dysregulated in both the C3 and C22 mouse models throughout their postnatal development when compared with their littermate controls. (**D**) The super pathway of cholesterol biosynthesis *z*-scores is plotted over the developmental time points at which data collection was conducted. Littermate control *z*-scores are equivalent to zero on the *y*-axis of the graph. (**E**) Heat map illustrating the temporal expression profiles of dysregulated lipid metabolism-related genes in the C3 and C22 mouse models throughout development. Scale bar in **A** = 100 µm. Black arrows in **C** and **E** represent developmental time points from 3 to 12 weeks in age, as displayed in **B**.

We previously observed that the C3 mice have postnatal myelination defects^25^ similar to what was described for the C22 mouse model.^24^ Using immunohistochemistry, the level of human PMP22 expression was clearly visible in the C22 mice and was subtly visible in the sciatic nerves of the C3 mouse models (Fig. 1A). To investigate the dose-dependent effect of *PMP22* overexpression, we analysed the transcriptome in the sciatic nerves throughout development (at 3, 5, 7, 9 and 12 weeks of age) using bulk RNA-seq (overview in Fig. 1B). Our results showed that cholesterol biosynthesis (Fig. 1C and D) and lipid metabolism (Fig. 1E) were the major dysregulated pathways in these CMT1A mouse models when compared with their age-matched WT littermates. Interestingly, cholesterol biosynthesis was progressively upregulated in C3 mice throughout development, whereas it was continuously repressed in C22 mice (Fig. 1C and D). From these data, we conclude that *PMP22* overexpression has a dose-dependent inhibitory effect on the cholesterol and lipid metabolism.

### *PMP22* overexpression causes abnormal lipid metabolism in the C3 mouse model

**Figure 2:**
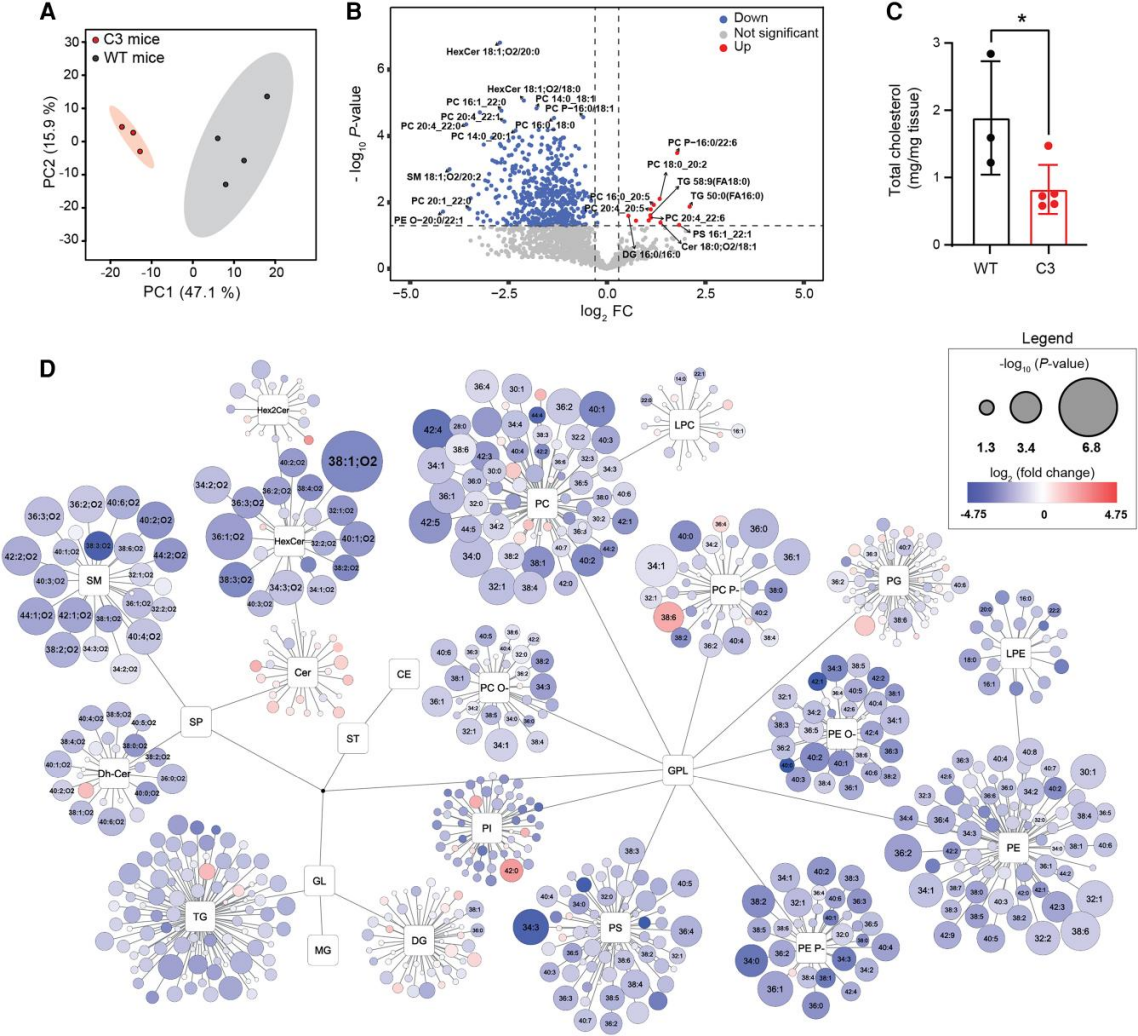
**Lipidomic analysis highlights major alterations in the expression of lipid species in the sciatic nerves of 5-week-old C3 mice**. (**A**) Principal component analysis (PCA) plot of lipids in sciatic nerves from C3 mice versus their wild-type (WT) littermate controls at 5 weeks of age. Principal components 1 and 2 (PC1 and PC2, respectively) were used to generate the graph. (**B**) Volcano plot demonstrating the relative expression of lipid species between C3 mice and WT controls. The *P*-value threshold was set at 0.05; fold change (FC) threshold: log_2_(FC) = 0.3. (**C**) Total cholesterol measurement from sciatic nerves of 5-week-old C3 mice and WT littermates. Statistical significance was evaluated using a two-tailed, unpaired *t*-test (*P < 0.05). Data are presented as the mean ± SD. (**D**) Simplified network visualization of lipid metabolism showing alterations in lipid profiles of C3 mice compared with their WT littermates. Circles represent the detected lipid species, where the circle size expresses the significance according to the *P*-value, while the colour darkness defines the degree of upregulation/downregulation (red/blue) according to the fold change. The most discriminating lipids are annotated. The number of mice used in **A**, **B** and **D** was as follows: WT mice = 4 and C3 mice = 3. In **C**, the number of mice used was as follows: WT mice = 3 and C3 mice = 5.

To investigate the role of PMP22 in cholesterol and lipid metabolism in more detail, we focused on the less severe C3 CMT1A mouse model. These C3 mice presented a dysmyelinating phenotype early in development, followed by an improvement in myelination.^24,25^ This correlates nicely with the aforementioned partial developmental amelioration in the cholesterol biosynthesis and lipid metabolism pathways (Fig. 1C and D). This resulted in thinner myelin sheaths and reduced axon calibre size in the peripheral nerves of adult mice, as previously described and quantified.^24^ At 5 weeks of age, we examined the lipid composition in the sciatic nerves of the C3 mice and compared it with that of their age-matched WT littermates. We observed a clear separation between the two mouse populations using PCA (Fig. 2A). A marked number of sphingolipids (SPs), especially sphingomyelin (SM), mono-hexosylceramide (HexCer), dihydroceramide (dhCer) and di-hexosylceramide (Hex2Cer), were significantly downregulated in C3 mice compared with WT controls (Fig. 2B). Contrary to the other SPs, most ceramide (Cer) lipids were upregulated in C3 mice, albeit without reaching statistical significance. Among the glycerolipids (GLs), the highly unsaturated triacylglycerols (TGs) were the most significantly downregulated. In the case of glycerophospholipids (GPL), the most significant alterations occurred in phosphatidylethanolamine (PE), PE plasmalogens (P-), PE ethers (O-), phosphatidylcholine (PC), PC plasmalogen (PC P-), PC ether (PC O-) and phosphatidylserine (PS). In addition, higher levels of long-chain (36, 38, 40, 42 and 44 total carbon number) and unsaturated PC species were observed, albeit without reaching statistical significance. Lysoglycerophospholipids [lysophosphatidylcholine (LPC) and lysophosphatidylethanolamine (LPE)], phosphatidylinositol (PI) and phosphatidylglycerol (PG) were mainly downregulated in the C3 mice, but only single lipids showed significant changes (Fig. 2B and D). Moreover, cholesterol levels were confirmed to be downregulated in the sciatic nerves of the C3 mouse model at this developmental time point (Fig. 2C), in line with the bulk RNA-seq results (Fig. 1C and D). Using a simplified lipid network, the sciatic nerve lipidome of the C3 mice was compared with the one of their WT littermates (Fig. 2D). Overall, these data confirm that lipid homeostasis is severely dysregulated in the sciatic nerves of the C3 CMT1A mouse model.

### CMT1A iPSC-SCPs show transcriptomic changes related to an abnormal lipid metabolism

**Figure 3:**
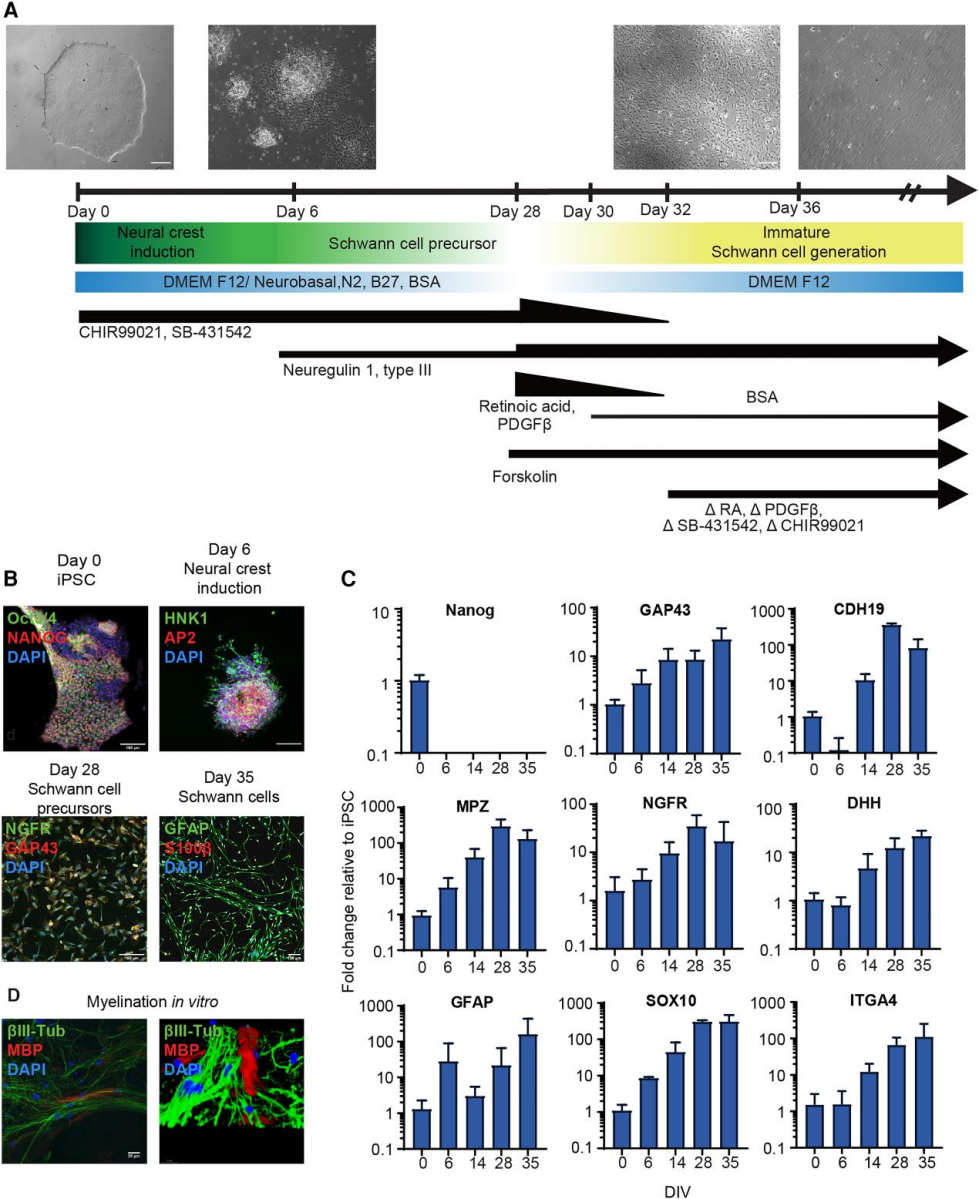
**Generation of Schwann cells and their precursors from induced pluripotent stem cells (iPSCs).** (**A**) Schematic overview of the iPSC-SC differentiation protocol, indicating incorporation or removal (Δ) of key medium components. Phase contrast images show the cultures at Days 0, 6, 28 and 36. Scale bar = 100 µm. (**B**) Immunocytochemical stainings of key lineage markers documenting a switch in phenotypes at Days 0 (iPSC; Nanog and Oct3/4), 6 (neural crest-like; AP2 and HNK1), 28 (SCP; NGFR and GAP43) and 35 (immature SC; GFAP and S100β) of the protocol. Scale bar = 50 µm. (**C**) Quantifications using qPCR of temporal mRNA expression of *NANOG*, *GAP43*, *CDH19*, *MPZ*, *NGFR*, *DHH*, *GFAP*, *SOX10* and *ITGA4*. DIV = days *in vitro*. Data are presented as the mean ± SD. (**D**) Immunocytochemical staining of iPSC-SCs showing expression of myelin basic protein (MBP) after being co-cultured with mouse dorsal root ganglia for 8 weeks. Scale bar = 20 µm. The image on the *right* shows a three-dimensional reconstruction using Leica SP8 confocal microscopy of the image on the *left*. Scale bar = 10 µm.

To investigate the effects of *PMP22* duplication in the context of CMT1A using a human-derived model, we used a protocol adapted from Kim *et al.*^29^ to generate iPSC-derived Schwann cell precursors (iPSC-SCPs) (Fig. 3A and Supplementary Fig. 2) and iPSC-derived Schwann cells (iPSC-SCs) (Fig. 3A) from a patient line, CS67i-CMT-n1, alongside its isogenic control iPSC line, isogenic-CS67i-CMT (referred to as ‘CMT1A’ and ‘isogenic’ lines, respectively) (Supplementary Figs 4 and 5). The isogenic line contained one copy of *PMP22* less, in comparison to its CMT1A mutant counterpart (Supplementary Fig. 6).

During differentiation, the iPSC colonies acquired a Schwann cell-like morphology, accompanied by the expression of key proteins marking the transitions from iPSCs to early neural crest cells, SCPs and, ultimately, Schwann cells (Fig. 3B and Supplementary Figs 2 and 3). Moreover, we documented the temporal expression profile of genes essential to Schwann cell lineage development (Fig. 3C). We also observed that these iPSC-SCs were capable of producing myelin when co-cultured with mouse dorsal root ganglion neurons (Fig. 3D).

**Figure 4:**
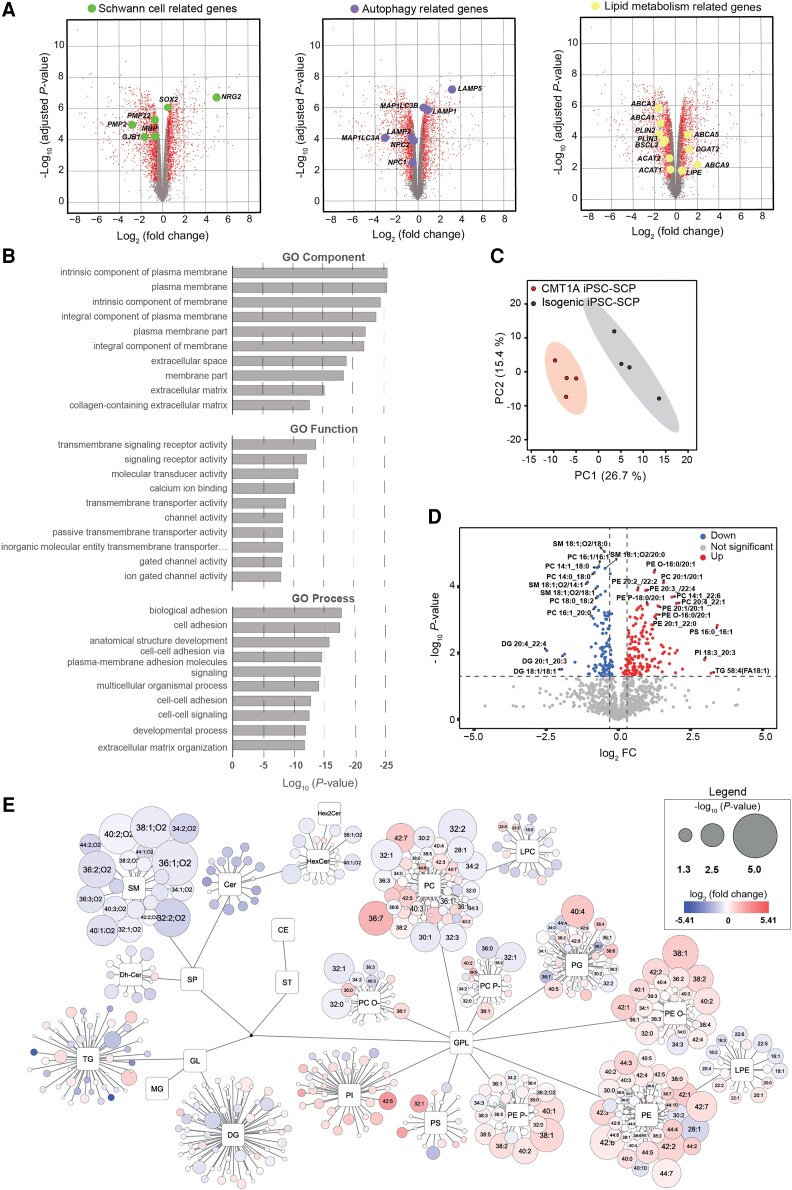
**Dysregulated gene expression and alterations of the lipidomic profile in CMT1A patient iPSC-SCPs.** (**A**) Volcano plots showing dysregulated genes in CMT1A iPSC-SCs compared with isogenic controls. Dysregulated myelin/Schwann cell-related genes, in addition to lipid metabolism- and autophagy-related genes are highlighted in separate graphs. The adjusted *P*-value threshold was set at 0.05; fold change (FC) threshold: 0.5. (**B**) Gene ontology (GO) showing the top 10 enriched ontological terms for cellular components (GO Component), function (GO Function) and molecular process (GO Process) that are reduced in CMT1A iPSC-SCPs. (**C**) Principal component analysis plot for the lipidomic analysis performed on CMT1A iPSC-SCPs and isogenic controls. (**D**) Volcano plot of the relative expression profiles of lipid species in the CMT1A iPSC-SCPs. The *P*-value threshold was set at 0.05; log_2_(FC) threshold: 0.3. (**E**) Simplified network visualization of lipid species, showing alterations in lipid profiles of CMT1A iPSC-SCPs compared with isogenic controls. Circles represent the detected lipid species, where the circle size expresses the significance according to the *P*-value, while the colour darkness defines the degree of upregulation/downregulation (red/blue) according to the fold change. The most discriminating lipids are annotated.

Bulk RNA-seq indicated that the transcriptome of CMT1A and isogenic iPSC-SCPs was similar to that of neural crest progenitors (Fig. 4A and Supplementary Fig. 7).^26^ We observed that several Schwann cell development and myelin-related genes, in addition to lipid metabolism and autophagy genes, were dysregulated in the CMT1A line in comparison to its isogenic iPSC-SCPs (Fig. 4A). Gene ontology (GO) enrichment analysis identified the PM component (GO component), receptor signalling and activity (GO function) and cell adhesion (GO process) as the core pathways enriched in CMT1A iPSC-SCPs (Fig. 4B). These data indicate early transcriptional dysregulation in the CMT1A iPSC-SCPs, altering the transcriptional networks of essential pathways for Schwann cell development, differentiation and maturation.

To understand how alterations in the transcriptome translate to lipid profiles in CMT1A, we performed a detailed lipidomic analysis on CMT1A and isogenic iPSC-SCPs. The PCA score plot, based on the measured lipid concentrations, showed a clear difference between CMT1A and isogenic iPSC-SCPs (Fig. 4C). Specifically, CMT1A iPSC-SCPs exhibited significantly lower levels of SM compared with the isogenic controls. Furthermore, CMT1A iPSC-SCPs had lower levels of Cer, HexCer and Dh-Cer but, in most cases, without reaching statistical significance. In the CMT1A iPSC-SCPs, many short-chain PC, PC P-, PC O-, PG, PE P- and PI were downregulated, whereas all short-chain PS were upregulated (Fig. 4D and E). We also detected a high accumulation of long-chain (38, 40, 42 and 44 total carbon number) and mostly unsaturated PE O-, PE and PE P-. Likewise, multiple long-chain and mostly unsaturated PC, PI and PG species were more abundant in CMT1A cells (Fig. 4D and E).

### CMT1A iPSC-SCPs have disordered plasma membranes with reduced lipid raft mobility

**Figure 5:**
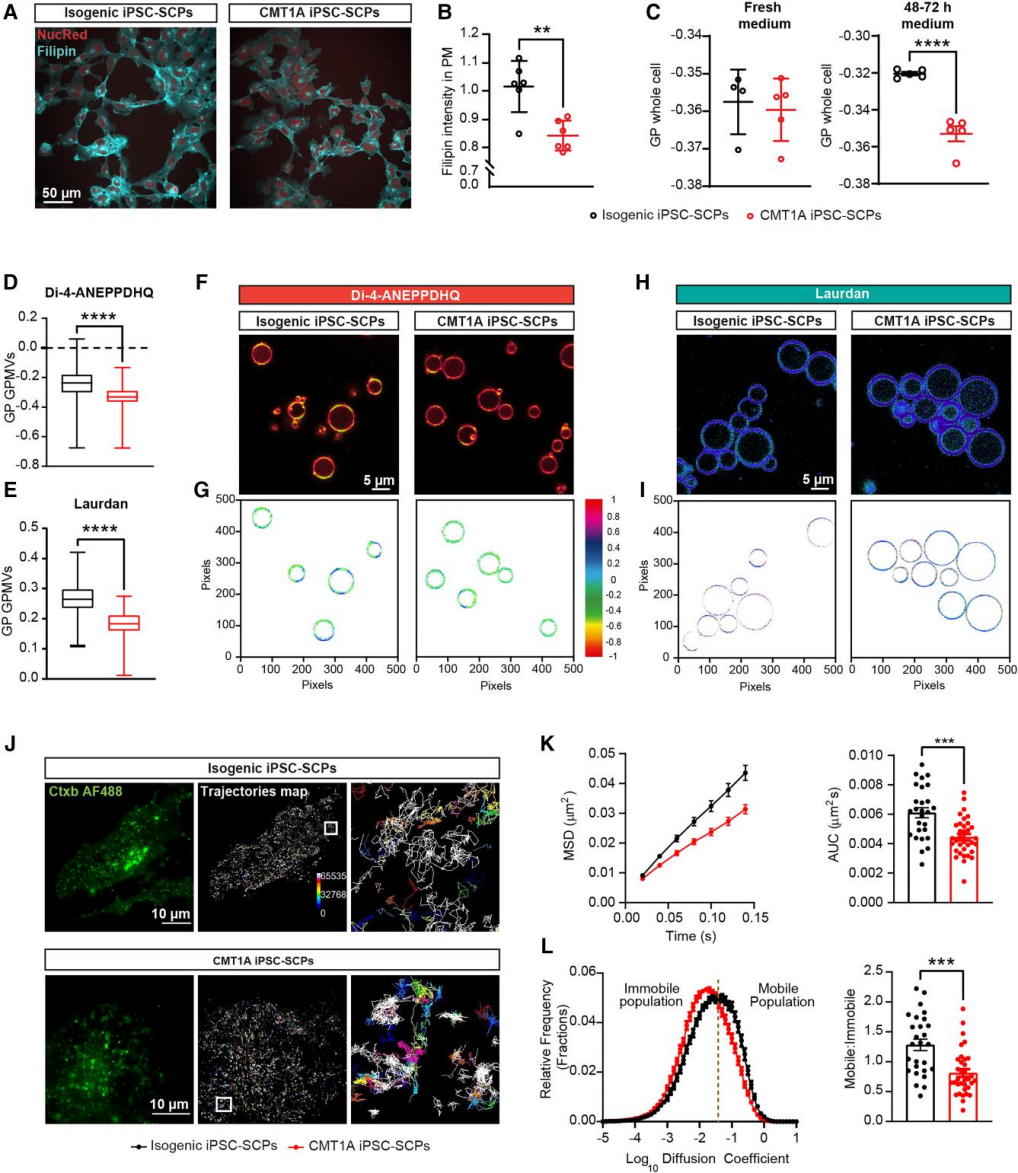
**Reduced free cholesterol, increased disorder and altered lipid raft dynamics in the plasma membrane of CMT1A iPSC-SCPs.** (**A**) Representative images of the filipin staining for isogenic and CMT1A iPSC-SCPs. (**B**) Free cholesterol levels in the plasma membrane of CMT1A iPSC-SCPs and isogenic controls were determined by filipin staining. *n* = average intensity per well, with ∼200 cells/well analysed. (**C**) Flow cytometry to calculate generalized polarization excitation (GP_ex_) values ranging from −1 (low membrane order) to +1 (high membrane order). *n* = individual wells cultured in parallel. (**D** and **E**) Spectral confocal imaging of giant plasma membrane vesicles (GPMVs) using Di-4-ANEPPDHQ (**D**) or Laurdan (**E**) to calculate the generalized polarization emmission (GP_em_) values. For Di-4-ANEPPDHQ, *n* = 164 CMT1A and *n* = 142 isogenic GPMVs, and for Laurdan, *n* = 151 CMT1A and *n* = 112 isogenic GPMVs. Representative figures are shown. (**F** and **G**) Di-4-ANEPPDHQ-stained GPMVs from CMT1A and isogenic iPSC-SCPs (**F**), colour-coded generalized polarization (GP) value per pixel (**G**). (**H** and **I**) Laurdan-stained GPMVs from CMT1A and isogenic iPSC-SCPs (**H**) colour-coded GP value per pixel (**I**). (**J**) Representative total internal reflection fluorescence images of CTB-labelled PM in isogenic and CMT1A iPSC-SCPs, and representative trajectory maps (*inset*: magnification exhibiting confined and free diffusion). (**K** and **L**) Mean square displacement (MSD) over time, area under the curve (AUC), mean distribution of the diffusion coefficient and the ratio of mobile to immobile fractions for CMT1A and isogenic iPSC-SCPs. Lipid raft dynamics is significantly confined in CMT1A. *n* = 27–34 cells, with 6966 trajectories per cell in the isogenic group and 5424 trajectories per cell in CMT1A group. Data are presented as the mean ± SD. **P < 0.01 and ****P < 0.0001.

Given that an altered lipid composition and accumulation of polyunsaturated fatty acids (PUFAs) might affect the fluidity of biological membranes and lipid raft organization and dynamics, we investigated the membrane properties in iPSC-SCPs. Using a filipin staining, a significant reduction in free PM cholesterol levels was observed in CMT1A iPSC-SCPs compared with their isogenic counterparts (Fig. 5A and B). Given that PM cholesterol preferentially interacts with lipids in liquid-ordered (L_o_) domains, such as in lipid rafts, we assessed membrane polarity using flow cytometry with the environmentally sensitive styryl dye Di-4-ANEPPDHQ.^33^ Interestingly, the membranes of CMT1A iPSC-SCPs became more liquid disordered (L_d_) when cultured in the same medium for 48–72 h, as opposed to a freshly changed medium (Fig. 5C). This could indicate that long-chain fatty acids and PUFAs accumulate in the membranes of CMT1A iPSC-SCPs over time.

To explore the PM specifically, spectral microscopic analysis was performed on Di-4-ANEPPDHQ-labelled GPMVs. CMT1A cells showed a significantly lower generalized polarization (GP) value compared with their isogenic controls (Fig. 5D, F and G), indicating a higher prevalence of L_d_ phases in CMT1A iPSC-SCPs, corroborating the flow cytometry data (Fig. 5C). Corresponding reduced GP values were observed in the membranes of CMT1A iPSC-SCPs using Laurdan (Fig. 5E, H and I). GP histograms indicate a decreased number of pixels with higher GP values in CMT1A compared with control GPMVs (Supplementary Fig. 8C and G). In addition, phasor plots, a model-free Fourier transform analysis (Supplementary Fig. 8D and H),^34-36^ demonstrate a significant shift in membrane ordering between isogenic and CMT1A GPMVs.

Next, PM fluidity was assessed in CMT1A patient-derived SCPs by studying lipid raft dynamics (Fig. 5J–L). Single-particle tracking revealed a notable reduction in lipid raft mobility in the PM of CMT1A iPSC-SCPs (Fig. 5K). Moreover, the ratio of mobile to immobile raft fractions indicated significantly confined dynamics of raft nanodomains in CMT1A (Fig. 5L). Total internal reflection fluorescence imaging of the cell membrane, along with trajectory maps illustrating the free hop-like diffusion, indicated a constrained movement of lipid raft domains in the PM of in CMT1A SCPs (Fig. 5J). These findings strongly suggest that alterations in the lipidome of CMT1A iPSC-SCPs disrupt the properties of the PM and lipid raft dynamics.

### Lipid homeostasis and autophagy are dysregulated during lipid stress in CMT1A iPSC-SCPs

**Figure 6:**
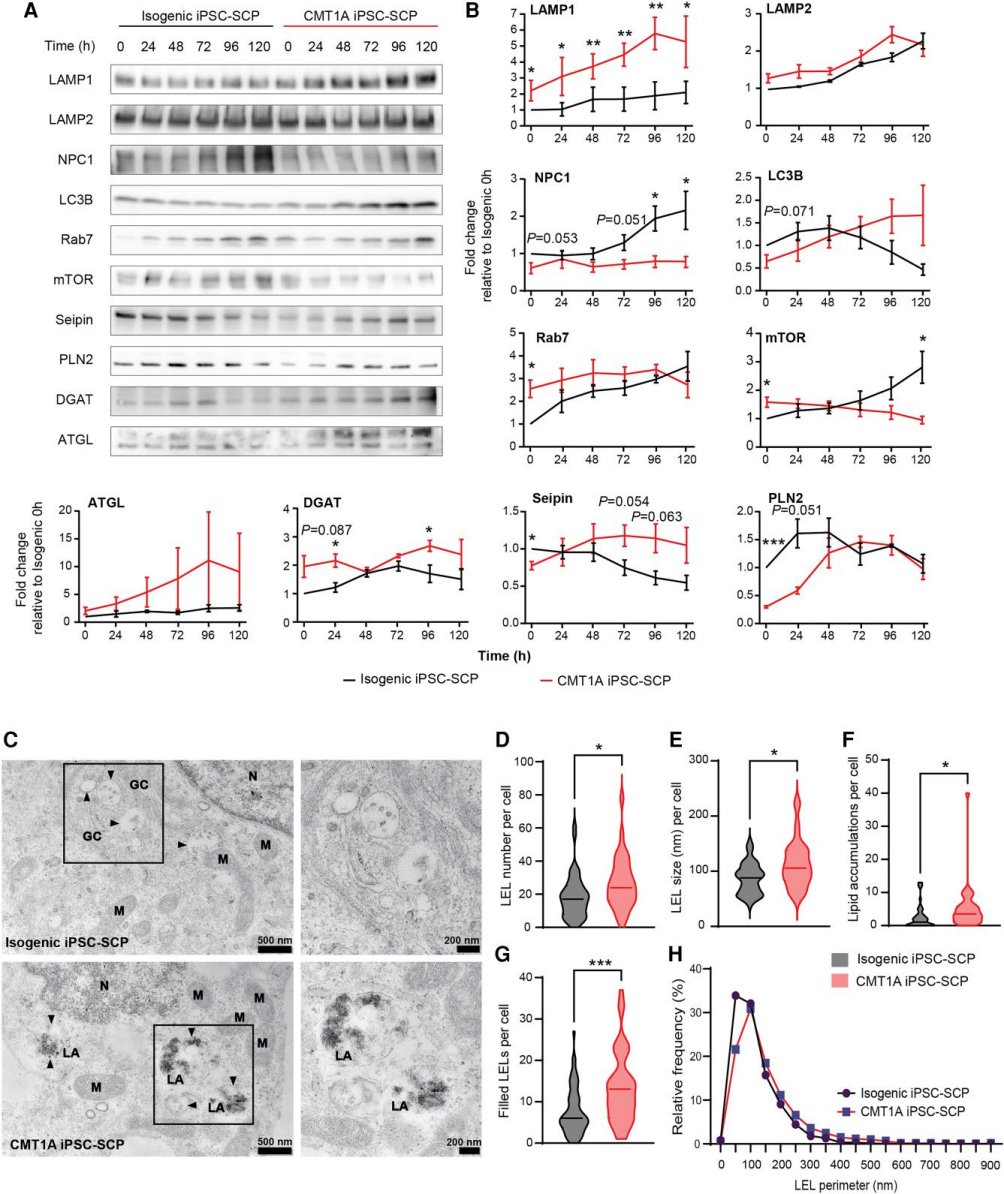
**Dysregulated lipid homeostasis and autophagy during lipid stress and lipid accumulations in the late-endosomal lysosomal system in CMT1A iPSC-SCPs.** (**A**) Immunoblots of key proteins of autophagy and lipid homeostasis in CMT1A iPSC-SCPs and isogenic controls during lipid starvation (0, 24, 48, 72, 96 and 120 h). (**B**) Quantification of blots displayed in **A**. Data are presented as the mean ± SEM. *n* = 3–5 independent time-course experiments. (**C**) Transmission electron microscopy images of CMT1A and isogenic iPSC-SCPs. GC = Golgi compartment; LA = lipid accumulation; M = mitochondria; N = nucleus. Black arrowheads indicate the perimeter of the vesicles. An enlarged area shows late-endosomal lysosomes (LELs) in isogenic iPSC-SCPs (*top*) and ‘filled’ LELs with lipid accumulations in CMT1A iPSC-SCPs (*bottom*). Scale bars = 500 nm for *left* images; 200 nm for *insets*. (**D**–**G**) Quantifications of LEL number (**D**) and size (**E**) per cell. Lipid accumulations per cell (**F**) present in LELs and non-vesicular bound accumulations. The number of ‘filled’ LELs (**G**), representing lipid and non-lipid accumulations in LELs that occupied at least one-fifth of the LEL. Isogenic iPSC-SCP cells = 45; CMT1A iPSC-SCP cells = 33. (**H**) Relative frequency distribution of the total number of LELs quantified in two independent preparations (isogenic iPSC-SCP LELs = 600; CMT1A iPSC-SCP LELs = 611). Statistical significance in **B** was evaluated using a two-way ANOVA, followed by Fisher’s LSD test (*P < 0.05, **P < 0.01 and ***P < 0.001). In **D**–**H**, statistical significance was evaluated using Student’s two-tailed, unpaired *t*-test (*P < 0.05 and ***P < 0.001).

Our lipidomics data showed that the lipid flux is altered in CMT1A. To understand how lipids are metabolized, we investigated the impact of prolonged lipid starvation on lipid homeostasis by monitoring autophagy, lipid biogenesis and lipolysis over time. iPSC-SCPs were lipid deprived by completely removing the BSA from the medium, and the following lysosomal/autophagy markers were assessed using western blot: lysosomal-associated membrane protein-1 (LAMP1), LAMP2 and microtubule-associated proteins 1A/1B light chain 3B (LC3B). The mammalian target of rapamycin (mTOR), a master regulator of autophagy and cellular response to stress and growth, and late endosome–lysosome (LEL) markers Rab7 and Niemann–Pick C1 protein (NPC1), involved in intracellular cholesterol handling and trafficking,^37-39^ were also examined. In CMT1A iPSC-SCPs, LAMP1 levels were consistently elevated, while LAMP2 expression remained similar to that of isogenic iPSC-SCP (Fig. 6A and B). LC3B was progressively upregulated in the CMT1A line, whereas in the isogenic line, it was initially upregulated and progressively downregulated (Fig. 6A and B). mTOR was initially upregulated in CMT1A iPSC-SCPs, similar to previous reports,^40^ but was downregulated during lipid starvation, whereas it was increased in the isogenic iPSC-SCPs. Rab7 was initially upregulated in CMT1A iPSC-SCPs but failed to mount the same response as the isogenic iPSC-SCPs over time. Interestingly, NPC1 was downregulated throughout the time-course experiment, which suggests alterations in lipid and cholesterol handling and trafficking in LELs in the CMT1A iPSC-SCPs (Fig. 6A and B).

We also investigated markers for LD biogenesis and lipolysis, by monitoring seipin, which is essential for LD biogenesis,^41,42^ DGAT1, which mediates the conversion of DG and fatty acid-CoA to TGs, perilipin 2 (PLN2) and ATGL. PLN2 is correlated with the size of LDs, because it is localized specifically on the LD membrane.^43^ During lipid starvation, LD biogenesis and size were delayed in the CMT1A iPSC-SCPs, while DGAT1 was slightly elevated at the start and at the end of the time course (Fig. 6A and B). ATGL displayed a trend of progressive upregulation in the CMT1A iPSC-SCPs (Fig. 6A and B).

Taken together, these data indicate that CMT1A iPSC-SCPs stimulate the autophagic response excessively and have a delayed LD-mediated response to lipid starvation.

### Lipids accumulate in the late endosome lysosomes in CMT1A iPSC-SCPs

Our findings could indicate a dysregulated intracellular lipid and cholesterol handling and trafficking, which might result in the accumulation of lipid products in the LELs. To investigate this, we performed transmission electron microscopy on CMT1A and isogenic iPSC-SCPs. Lipid accumulations were prominently visible in LELs of CMT1A iPSC-SCPs compared with controls (Fig. 6C, F and G). Furthermore, the number and size of LELs were significantly increased in CMT1A cells (Fig. 6D, E and H). This suggests that lipid trafficking and handling in LELs are perturbed in CMT1A iPSC-SCPs.

### Lipid droplets are generated excessively in CMT1A iPSC-SCPs

**Figure 7:**
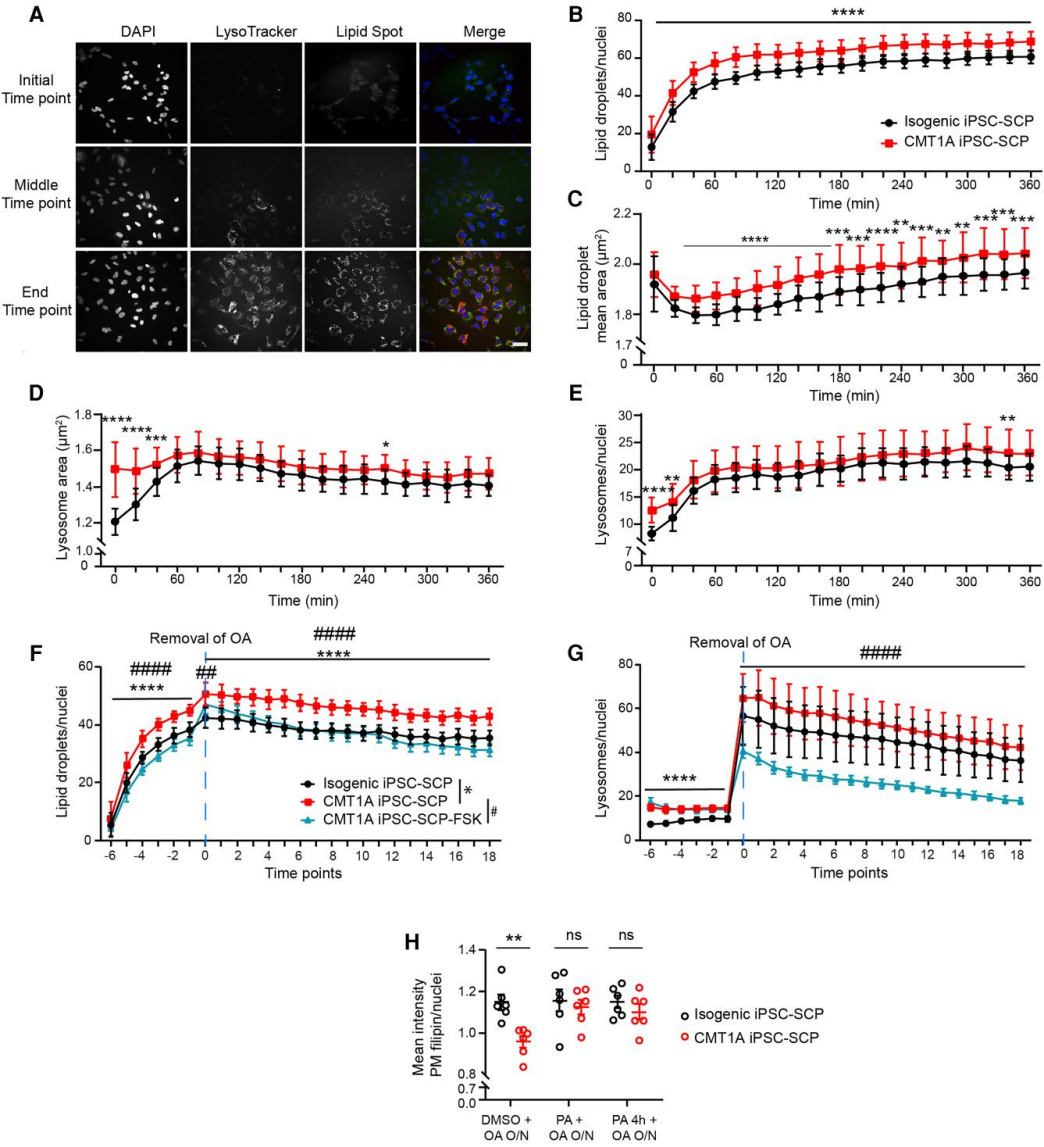
**Excessive lipid droplet formation in response to oleic acid exposure in CMT1A iPSC-SCPs, which can be reversed by modulating lipid metabolism.** (**A**) Immunofluorescence of lysosomes (LysoTracker, in red) and lipid droplets (LDs, LipidSpot, in green) of CMT1A iPSC-SCPs treated with oleic acid (OA) at the beginning, middle and end of the time-course experiment. Scale bar = 20 µm. (**B**–**E**) Analysis of the time-course experiment demonstrating higher LD numbers (**B**) and size (**C**) in CMT1A than in isogenic iPSC-SCPs. Lysosome size (**D**) and number (**E**) were initially increased in the CMT1A iPSC-SCPs, but then reached the level of isogenic iPSC-SCPs. Thirty wells per group were analysed. Data are presented as the mean ± SD. (**F** and **G**) Time-course imaging of lysosomes and LDs demonstrated that forskolin (FSK) prevented the excessive accumulation of LDs (**F**) and lysosomes (**G**) in CMT1A iPSC-SCPs during exposure to OA. Data are presented as the mean ± SD. (**H**) Treatment with progesterone receptor antagonist (PA) PF-02413873 resulted in increased free cholesterol in the plasma membrane. Data are presented as the mean ± SEM; 30 wells per group were analysed for both isogenic and CMT1A iPSC-SCPs. Analysis in **B**–**E** was performed using a one-way ANOVA (**P < 0.01, ***P < 0.001 and ****P < 0.0001; data are presented as the mean ± SD), in **F** and **G** using a two-way ANOVA with Šidák multiple comparisons tests (****^,####^*P* < 0.0001; data are presented as the mean ± SD), and in **H** by using Student’s two-tailed *t*-test (**P < 0.01, ***P < 0.001 and ****P < 0.0001).

Given that lipid storage and homeostasis are dysregulated in CMT1A iPSC-SCPs, we investigated how these patient-derived cells mobilize lipids during short-term lipid abundance. Therefore, we monitored the response of CMT1A iPSC-SCPs to the induction of LD formation by treating them with oleic acid (OA), a stimulator of lipid droplet generation. The generation of LDs and lysosomes was monitored over 7 h using LipidSpot and LysoTracker (Fig. 7A). Interestingly, the number and size of LDs were significantly increased in the CMT1A iPSC-SCPs in comparison to their isogenic control (Fig. 7B and C). Additionally, the size and number of lysosomes were higher at the 0 h time point (Fig. 7D and E), but then normalized in comparison to the isogenic iPSC-SCPs during continuous OA treatment. These data not only confirm that the size and number of LELs were increased in the CMT1A iPSC-SCPs, but the response of the CMT1A iPSC-SCPs to OA in the size and production of LDs also strongly indicates that one additional copy of *PMP22* causes abnormal lipid storage and lipid-mediated stress.

### Stimulation of autophagy and lipolysis restores lipid homeostasis in CMT1A iPSC-SCPs

To restore the lipid metabolic phenotypes, we modulated lipolysis and autophagy in CMT1A and isogenic controls. Therefore, we treated CMT1A iPSC-SCPs with FSK, a cAMP activator and a potent stimulator of lipolysis and autophagy.^44-46^ FSK reduced LD generation in the presence of OA (Fig. 7F). Moreover, the decline in LDs was similar between the CMT1A and isogenic iPSC-SCPs after OA removal. However, FSK continued to stimulate LD breakdown (Fig. 7F). Although FSK did not strongly affect the number of lysosomes in the presence of OA, the number of lysosomes decreased significantly in FSK-treated CMT1A iPSC-SCPs after OA removal (Fig. 7G).

**Figure 8:**
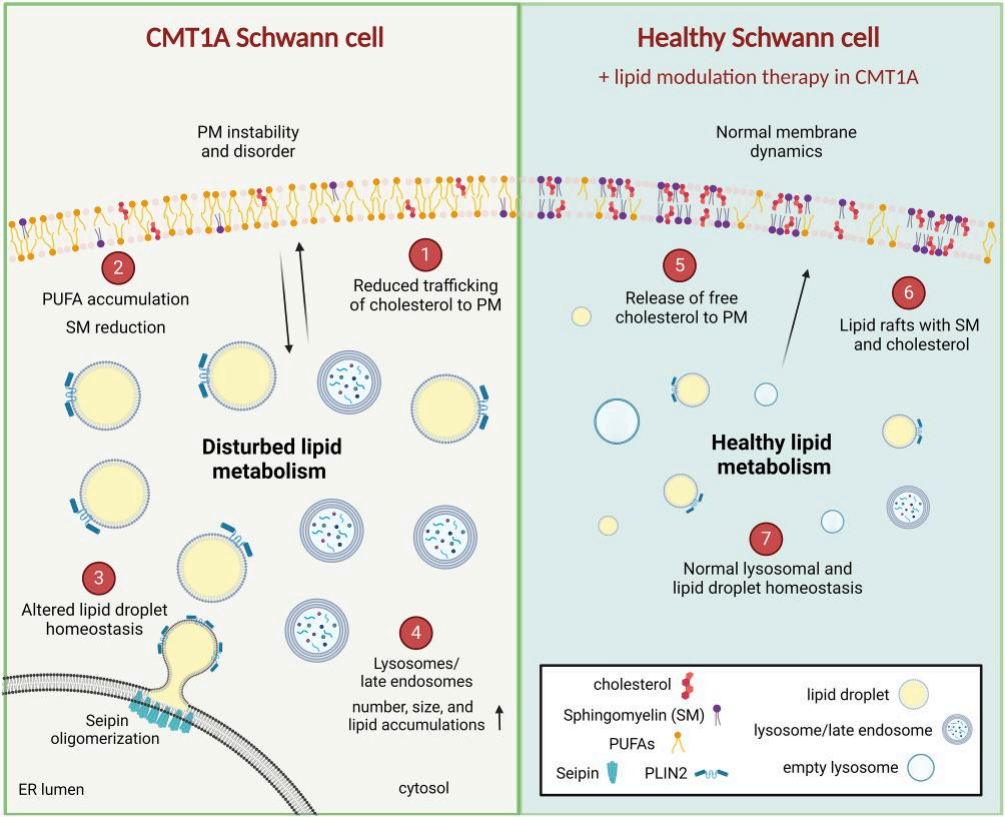
**Schematic overview of the perturbed lipid homeostasis during human CMT1A Schwann cell development.** Homeostatic regulation of lipid metabolism is perturbed during the development of human CMT1A Schwann cells. Illustration of how human CMT1A Schwann cells respond to lipid abundance and starvation, including (**1**) reduced cellular trafficking and incorporation of cholesterol; and (**2**) excess accumulation of polyunsaturated fatty acids (PUFAs) and reduction of sphingomyelin (SM) in the plasma membrane (PM). These alterations in PM lipid composition cause the PM to become disordered, which perturbs lipid raft functions and lipid-mediated signalling, thereby affecting the stability of the PM and cell differentiation state in CMT1A Schwann cells. In addition, CMT1A Schwann cells displayed alterations in (**3**) lipid droplet biogenesis, size and number during nutrient stress; and, likewise, (**4**) the number and size of lysosomes and late endosomal lysosomes (LELs), and the number of lipid-loaded LELs was increased. Importantly, treatment strategies targeting the lipid droplets were shown effectively to (**5**) release the storage of free cholesterol and enable its incorporation into the PM; and (**6**) stabilize the PM and lipid raft dynamics. (**7**) Lastly, the targeting of perturbed lipid droplets and lysosome regulation can restore the lipid homeostatic balance in human CMT1A Schwann cells. This figure was created with BioRender.com.

Next, we stimulated free cholesterol incorporation into the PM of CMT1A iPSC-SCPs using a progesterone receptor antagonist known as PF-02413873. Progesterone itself is synthesized from cholesterol in a two-step process^47^ and acts as an inhibitor of cholesterol biosynthesis.^48^ Treatment with a progesterone receptor antagonist for 4 h or overnight rescued the PM cholesterol deficit in the CMT1A iPSC-SCPs (Fig. 7H). This implies that selectively targeting cholesterol release from LDs facilitates the incorporation of free cholesterol into the PM of CMT1A iPSC-SCPs (summarized in Fig. 8).

## Discussion

CMT1A pathophysiology is characterized by a slowly progressive dysmyelinating phenotype, starting from early development, that leads to axonal loss, denervation of neuromuscular junctions and sensory problems. We used a combination of CMT1A mouse models and patient-derived iPSC-SCPs to investigate how pathological alterations in lipid metabolism cause CMT1A. We identified cholesterol biosynthesis and lipid metabolism as the main canonical pathway downregulated in the sciatic nerves of both the C3 and C22 mouse models, similar to what was reported in CMT1A rats.^49^ The *PMP22* copy number had a dose-dependent effect on cholesterol biosynthesis, which we found by comparing the C3 and C22 mouse models, which express five and seven copies of the human *PMP22* gene, respectively. High cholesterol levels are essential for myelin membrane growth.^50^ Likewise, *PMP22* overexpression reduced the expression of genes regulating the lipidome transcriptional network in a dose-dependent manner. In the outbred rat model of CMT1A,^22^ rats can have varying levels of rodent *Pmp22* expression, giving rise to a spectrum of phenotype severities, including variations in the expression of lipid metabolism-related genes.^22,49^ To understand how these transcriptional changes influence the CMT1A lipid profiles, we performed lipidomics on the sciatic nerves of 5-week-old C3 mice. Overall, the analysis revealed a major downregulation of lipid species in general, particularly of SPs, with SMs being the most affected in the sciatic nerves of C3 mice compared with their age-matched littermate controls. In further support of these alterations, CMT1A rat sciatic nerve myelin exhibited significantly reduced levels of total cholesterol, PC, PE, plasmalogens, SM and ceramides.^49^

The correct PM lipid composition is essential for Schwann cells to interact with axons and to produce myelin, the multilamellar lipid-based structure. During Schwann cell lineage development, neural crest cells give rise to SCPs, which are guided to the most distal regions of the body by the extending peripheral axons.^51^ Once interacting with axons, SCPs can differentiate into immature Schwann cells, which organize axons to be myelinated and non-myelinated in a process defined as radial sorting.^52^ To investigate the cellular origins of CMT1A lipid alterations, we developed a protocol to derive a Schwann cell lineage from iPSCs. These differentiated iPSC-SCs had Schwann cell bi- and tripolar morphologies, expressed Schwann cell genes and showed myelination *in vitro*. However, myelination was seen only at an extremely low frequency and could not be used reliably for myelination assays in co-cultures with rodent neurons.

Given that differentiation defects in the development of mature Schwann cells have been described in CMT1A rodents^53^ and iPSCs,^54^ we hypothesized that early cellular pathologies originate in the CMT1A iPSC-SCPs. SCPs represent a premyelinating cell source of the Schwann cell lineage expressing PMP22.^51^ This allows us to study the molecular mechanisms of PMP22 in the early stages of Schwann cell development, prior to the onset of myelination, and to explore how this is affected in CMT1A. Moreover, the iPSC-SCP model offers a homogeneous and proliferating cell source of the human Schwann cell lineage, which allows high-throughput screening and omics analysis in a controlled two-dimensional environment.

Transcriptional analysis of CMT1A patient iPSC-SCPs showed a downregulation of several genes related to Schwann cell myelination and development. In addition, key lipid metabolism genes were downregulated, including the cholesterol efflux transporters ATP binding cassette subfamily A 1 (ABCA1) and ABCA3. PMP22 is known to regulate ABCA1-mediated cholesterol efflux, because *Pmp22* knockout drastically upregulated ABCA1 and led to LD accumulations in the peripheral nerves.^17^ In addition, Zhou *et al.*^55^ showed cholesterol sequestration to lysosomes and reduced ATP-binding cassette transporter-dependent cholesterol efflux in CMT1A patient fibroblasts. In CMT1A iPSC-SCPs, we showed that several genes relating to lipid homeostasis and storage were downregulated. Interestingly, the autophagy-associated genes *LAMP1* and *LAMP2* were dysregulated, whereas *NPC1* and *NPC2* were downregulated. NPC1 and NPC2 are intracellular transporters that act in tandem to remove cholesterol from the lysosomal compartment^37,39,56^ and regulate cholesterol homeostasis through the generation of low-density lipoprotein cholesterol-derived oxysterols.^56^ Loss of function of NPC1 or NPC2 proteins is known to cause the lysosomal storage disease Niemann–Pick disease, type C1 and 2, respectively.^37^

GO enrichment analysis revealed dysregulation in genes regulating PM components, transmembrane signalling receptor activity and biological adhesion, all of which are essential factors regulating axo-glial interactions and myelination maintenance.^57,58^

Lipidomic analysis showed significant alterations in SP in CMT1A iPSC-SCPs. Specifically, we observed a reduction in SM and an increase in Cer and lipids containing long acyl chain unsaturated fatty acids (e.g. PE O-, PE, PE P-, PC, PI and PG). These alterations might offer insights into the functional deficits observed in Schwann cells in CMT1A. Maintaining the correct PM lipid composition, along with high cholesterol levels,^50,59^ is essential for Schwann cell signalling and to interact and bend around axons, while producing myelin.

Given that the storage and release of cholesterol or PUFAs from LDs can influence the ratio of ordered and disordered domains in the PM,^60^ we investigated PM changes in CMT1A patient-derived cells. We identified a reduction in PM cholesterol levels and found a progressive shift towards a disordered state over time. Considering that lipid rafts, specialized microdomains within the PM, rely on a balanced interaction between cholesterol and SM, these data suggest impairment in lipid raft-mediated signalling and lipid transport. This impairment might lead to an increase in PM disorder and the accumulation of PUFAs in CMT1A.

Lipid rafts are crucial for regulating the activity of membrane receptors and signalling molecules, as evident from GO enrichment analysis in CMT1A iPSC-SCPs. These specialized domains are characterized by high levels of saturation, cholesterol and SPs, rendering them resistant to thermal and chemical perturbations.^61^ We demonstrated decreased levels of cholesterol and SM, along with an increase in PM disorder, in CMT1A patient-derived iPSC-SCPs and mouse models. These changes disrupt the normal lipid–protein balance within lipid raft domains, affecting the function of membrane receptors. As a consequence, this disruption might result in altered receptor signalling and myelination. Using live lipid raft tracking, we confirmed these alterations in the dynamics of lipid rafts in the PM of CMT1A iPSC-SCPs, impacting PM fluidity.

Of note, the Cer–SM balance can affect receptor-mediated signal transduction and the integrity of lipid rafts.^62^ Sphingomyelinase can stimulate the conversion of SM into Cer, which, unlike SM, creates a negative electrical environment for cholesterol, facilitating its removal from the membrane. This implies that changes in lipid raft formation can impact cholesterol metabolism, and vice versa^62,63^ Our lipidomics results demonstrate a significant decrease in SM in both human CMT1A iPSC-SCPs and CMT1A mice, indicating an impairment in SM metabolism. Interestingly, PMP22 has a known preference for partitioning to cholesterol-rich L_o_ phase domains^64^ and is known to be essential for the formation of lipid rafts,^16^ indicating its regulatory significance. Notably, similar dysregulations of cholesterol transport and incorporation in the plasma membrane affecting lipid rafts have been observed in animal models of related neuropathies. These include the hereditary neuropathy with liability to pressure palsies mouse models with *Pmp22* haploinsufficiency and the TremblerJ mouse models harbouring a point mutation in *Pmp22*.^17,65^ These findings, along with other studies, confirmed the role of PMP22 as a protein that can directly stabilize ordered-phase membrane domains.^66^ Taken together with our results, these studies suggest that the effects of PMP22 on lipid composition are likely to result from its dysregulation and are not indicative of a gain-of-function mechanism.

Considering that the lipid composition undergoes substantial changes during postnatal development, our data indicate that *PMP22* duplication impacts specific lipid classes, mostly SPs, leading to dysregulation of the membrane and lipid storage. These changes might contribute to the onset of CMT1A by altering the initiation of myelination or the stability of its structure. Interestingly, modulating the lipid pathway, and specifically SP, has been shown to have positive effects *in vitro* on CMT1A rodent cultured neurons.^67^ Moreover, changes in SP metabolism have been discovered to be not only limited to the myelin, but can also be found in the biological fluids of CMT1A patients, suggesting a systemic metabolic dysfunction.^67^

Consistent with our findings from lipidomic and transcriptomic analysis, we observed a delay in LD growth and biogenesis as a response to starvation, indicating an impaired lipid mobilization in CMT1A iPSC-SCPs. Notably, mTOR, a master metabolic regulator of cellular responses to nutrient availability, was initially upregulated in basal conditions, similar to what has been reported recently.^40^ Interestingly, mTOR was progressively downregulated throughout the course of lipid starvation, whereas it increased progressively in the isogenic iPSC-SCPs.

Furthermore, markers such as LAMP1 and LC3B, associated with late endosomes/lysosomes and autophagy, respectively, were excessively stimulated in CMT1A iPSC-SCPs. Strikingly, in CMT1A iPSC-SCPs, NPC1 was downregulated and failed to mount a response to lipid starvation similar to what was observed in the isogenic iPSC-SCPs. This suggests the possibility of lipid accumulation in LELs. Transmission electron microscopy corroborated these findings, revealing increased LEL size and number, with excessive lipid accumulations in the CMT1A iPSC-SCPs compared with isogenic control iPSC-SCPs.

Alterations in lipid handling and storage might affect the trafficking of lipids to cellular membranes. In yeast, catabolism of LDs regulates rapid membrane expansion.^68^ To understand how CMT1A iPSC-SCPs handle an excess of lipids, we studied the generation of LDs during OA treatment. Surprisingly, CMT1A iPSC-SCPs presented an excessive LD production and enlargement in response to OA exposure. In line with our transmission electron microscopy data, the size and number of lysosomes were increased at the start of OA exposure in CMT1A iPSC-SCPs but then normalized to the levels observed in isogenic control cells. Collectively, these data indicate a deficiency in lipid handling and autophagy in CMT1A patient cells.

Lastly, to validate these lipid-related phenotypes as a therapeutic target, we modulated the phenotype of LDs using FSK, a cAMP stimulator known for activating lipolysis,^44^ autophagy^45,46^ and myelin gene expression.^69,70^ In CMT1A iPSC-SCPs, FSK successfully mitigated excessive LD production induced by OA. When OA was removed from the cell culture medium, FSK significantly reduced the number of lysosomes per cell, demonstrating a beneficial effect. As a proof of concept, we also used a progesterone receptor antagonist, a known inhibitor of *PMP22* expression^71,72^ and a stimulator of free cholesterol release,^48^ to modulate the cholesterol deficit in CMT1A iPSC-SCPs. After both a short and an overnight incubation, we found that free cholesterol levels were restored in the PM of CMT1A iPSC-SCPs, indicating a positive outcome of the treatment.

## Conclusion

In conclusion, we demonstrated that disruptions in cellular lipid homeostasis in CMT1A originate at an early stage in the Schwann cell lineage. Moreover, these lipid imbalances can influence the regulation of lipid storage, thereby affecting the availability of lipids and cholesterol within the PM. The dysregulation of membrane lipids causes an increased incorporation of PUFAs into the PM, resulting in PM disorder and impairing lipid raft dynamics. Furthermore, we have demonstrated the critical relationship between membrane lipids and lipid storage homeostasis, which is significantly perturbed in CMT1A iPSC-SCPs. Our findings, summarized and illustrated in Fig. 8, highlight the pivotal role of PMP22 in regulating PM lipid composition and lipid storage homeostasis early in Schwann cell development. Future investigations could explore these findings in more complex iPSC-derived models, including peripheral nerve organoids^73^ and mature Schwann cells, alone or in co-culture with neurons, to elucidate comprehensively the function of PMP22 and the consequences of its alterations in CMT1A.

Our data emphasize that targeting lipid metabolic pathways holds promise as a potential therapeutic^74–76^ intervention for CMT1A patients. Different therapeutic approaches are possible to stimulate lipid metabolic pathways in CMT1A patients for improving myelination.^77,78^ These interventions include: (i) dietary lipid supplementation tailored to individual metabolic needs and clinical response; drugs stimulating (ii) cellular lipid uptake or (iii) endogenous lipid production; or (iv) those modulating intracellular lipid storage or trafficking. Given the regulatory role of PMP22 in cholesterol trafficking, lipid rafts and membrane stability, it is expected that *PMP22*-modulating therapies^75,76^ will also positively impact the biophysical properties of the Schwann cell plasma membrane and myelination.

Disturbances in lipid metabolism are implicated in various other peripheral neuropathies, including peripheral nerve trauma^78,79^ and other types of CMT, such as 1B,^80,81^ 1E^82^ and HNPP,^55^ amongst several others. This suggests that our findings extend to other dysmyelinating neuropathies in which lipid metabolism is affected, highlighting the broader relevance of our study.

## Supplementary Material

2479-Prior-et-al.-Revised-supplementary-section

## Data Availability

Data are available from the corresponding authors on reasonable request.
