# Supplementary material for: PMP22 duplication dysregulates lipid homeostasis and plasma membrane organization in developing human Schwann cells
Source: Brain. 2024 May 14;147(9):3113–30. doi: 10.1093/brain/awae158 (PMC11370802; doi:10.1093/brain/awae158)
Supplement: 2479-Prior-et-al.-Revised-supplementary-section [file 2479-prior-et-al.-revised-supplementary-section.docx]

**Supplementary information**

***PMP22* duplication dysregulates lipid homeostasis and plasma membrane organization in developing human Schwann cells**

Robert Prior,^1,2,3,†^ Alessio Silva,^1,2,†^ Tim Vangansewinkel,^2,4,†^ Jakub Idkowiak,^5,6^ Arun Kumar Tharkeshwar,^1,2^ Tom P. Hellings,^7^ Iliana Michailidou,^7^ Jeroen Vreijling,^7^ Maarten Loos,^8^ Bastijn Koopmans,^8^ Nina Vlek,^8^ Cedrick Agaser,^9^ Thomas B. Kuipers,^9^ Christine Michiels,^1,2^ Elisabeth Rossaert,^1,2^ Stijn Verschoren,^1,2^ Wendy Vermeire,^1,2^ Vincent de Laat,^5^ Jonas Dehairs,^5^ Kristel Eggermont,^1,2^ Diede van den Biggelaar,^1,2^ Adekunle T. Bademosi,^10^ Frederic A. Meunier,^10,11^ Martin vandeVen,^4^ Philip Van Damme,^1,2,12^ Hailiang Mei,^9^ Johannes V. Swinnen,^5^ Ivo Lambrichts,^4^ Frank Baas,^7^ Kees Fluiter,^7^ Esther Wolfs^4,‡^ and Ludo Van Den Bosch^1,2,‡^

**^†,‡^These authors contributed equally to this work.**

^1^KU Leuven - University of Leuven, Department of Neurosciences, Experimental Neurology and Leuven Brain Institute (LBI), Leuven, Belgium

^2^VIB, Center for Brain & Disease Research, Laboratory of Neurobiology, Leuven, Belgium

^3^Department of Ophthalmology, Medical Faculty, University of Bonn, Bonn, Germany
^4^UHasselt - Hasselt University, Biomedical Research Institute, Diepenbeek, Belgium

^5^Laboratory of Lipid Metabolism and Cancer, Department of Oncology, KU Leuven, Leuven, Belgium
^6^Department of Analytical Chemistry, Faculty of Chemical Technology, University of Pardubice, Pardubice, Czech Republic
^7^Department of Clinical Genetics, Leiden University Medical Center, Leiden, The Netherlands
^8^InnoSer Nederland B.V., Leiden, The Netherlands
^9^Department of Biomedical Data Sciences, Sequencing Analysis Support Core, Leiden University Medical Center, Leiden, The Netherlands
^10^Clem Jones Centre for Ageing Dementia Research, Queensland Brain Institute, The University of Queensland, Brisbane, Queensland, Australia
^11^School of Biomedical Sciences, The University of Queensland, Brisbane, Queensland, Australia
^12^University Hospitals Leuven, Department of Neurology, Leuven, Belgium

**List of Abbreviations**

| ABC | ATP-binding cassette transporters |
| --- | --- |
| ACAT2 | Acetyl-CoA acetyltransferase 2 |
| ACTB | ꞵ-actin |
| Ara-c | Cytosine arabinoside |
| ATGL | Adipose triglyceride lipase |
| AUC | Area under the MSD curve |
| ΒIII-TUB | βIII-tubulin |
| BSA | Bovine serum albumin |
| C3 | C3-PMP22 mouse model |
| C22 | C22-PMP22 mouse model |
| cAMP | Cyclic adenosine monophosphate |
| CDH19 | Cadherin 19 |
| Cer | Ceramide |
| CMT | Charcot-Marie-Tooth disease |
| CMT1A | Charcot-Marie-Tooth disease type 1A |
| CTB | Cholera toxin b |
| ddPCR | Digital droplet PCR |
| DHH | Desert hedgehog |
| DRG | Dorsal root ganglion |
| DG | Diacylglycerol |
| DGAT1 | Diacylglycerol acyltransferase 1 |
| EGR2/KROX-20 | Early growth response 2 |
| FSK | Forskolin |
| GAP43 | Growth associated protein 43 |
| GFAP | Glial fibrillary acidic protein |
| GL | Glycerolipids |
| GO | Gene ontology |
| GP | Generalized polarization |
| GPL | Glycerophospholipids |
| GPMVs | Giant plasma membrane vesicles |
| Hex2Cer | Di-hexosylceramides |
| HexCer | Mono-hexosylceramides |
| HNK-1 | Human Natural Killer 1 |
| HNPP  iPSC | Hereditary Neuropathy with liability to Pressure Palsies  Induced pluripotent stem cells |
| iPSC-SCs | Induced pluripotent stem cell-derived Schwann cells |
| iPSC-SCPs | Induced pluripotent stem cell-derived Schwann cell precursors |
| ITGA4 | Integrin subunit alpha 4 |
| LAMP1 | Lysosomal-associated membrane protein-1 |
| LAMP2 | Lysosomal-associated membrane protein-2 |
| LC3B | Microtubule-associated proteins 1A/1B light chain 3B |
| LD | Lipid droplet |
| L_d_ | Liquid-disordered phase |
| LEL | Late endosome-lysosome |
| L_o_ | Liquid-ordered phase |
| LPC | Lysophosphatidylcholines |
| LPE | Lysophosphatidylethanolamines |
| LSD | Least significant difference |
| MBP | Myelin basic protein |
| MPZ | Myelin protein zero |
| MSD | Mean square displacement |
| mTOR | Mammalian target of rapamycin |
| NGFR | Nerve growth factor receptor |
| NPC1 | Niemann–Pick C1 protein |
| NRG1, type III | Neuregulin-1, type III |
| OA | Oleic acid |
| OCT3/4 | Octamer-binding transcription factor 3/4 |
| PBS | Phosphate buffer saline |
| PC | Phosphatidylcholines |
| PCA | Principal component analysis |
| PC O- | Phosphatidylcholine ethers |
| PC P- | Phosphatidylcholine plasmalogens |
| PDGFβ | Platelet-derived growth factor subunit β |
| PE | Phosphatidylethanolamines |
| PE O- | Phosphatidylethanolamine ethers |
| PE P- | Phosphatidylethanolamine plasmalogens |
| PG | Phosphatidylglycerols |
| PI | Phosphatidylinositols |
| PLN2 | Perilipin 2 |
| PM | Plasma membrane |
| PMP22 | Peripheral myelin protein 22 (human) |
| Pmp22 | Peripheral myelin protein 22 (mouse) |
| PNS | Peripheral nervous system |
| PUFAs | Polyunsaturated fatty acids |
| RA | Retinoic acid |
| Rab7 | Ras-related protein 7 |
| RT | Room temperature |
| S100β | S100 calcium-binding protein β |
| SCP | Schwann cell precursor |
| SM | Sphingomyelins |
| SP | Sphingolipids |
| SOX2 | Sex determining region of Y-related high mobility group box 2 |
| SOX10 | Sex determining region of Y-related high mobility group box 10 |
| TFAP2 | Transcription factor AP-2α |
| TG | Triacylglycerol |
| TIRF | Total internal reflection fluorescence |
| WT | Wild-type |

**Supplementary Tables**

**Table S1: Primers used for qPCR analysis.**

| Gene | Direction | Primer sequence |
| --- | --- | --- |
| 18S | Forward  Reverse | 5’ – GGC CCT GTA ATT GGA ATG AG – 3’  5’ – GCT ATT GGA GCT GGA ATT AC – 3’ |
| ACTB | Forward  Reverse | 5’ – CAT GTA CGT TGC TAT CCA GGC– 3’  5’ – CTC CTT AAT GTC ACG CAC GAT – 3’ |
| CDH19 | Forward  Reverse | 5’ – ATT GGT CAG CCA GGA GCG TTG T – 3’  5’ – GCA GAT TCA GAG ACA GTC AAG CG – 3’ |
| DHH | Forward  Reverse | 5’ – AGG ATG AGG AGA ACA GTG GAG C – 3’  5’ – TCA GTC ACT CGT AGG CGC ACT C – 3’ |
| GAP43 | Forward  Reverse | 5’ – GGC CGC AAC CAA AAT TCA GG – 3’  5’ – CGG CAG TAG TGG TGC CTT C – 3’ |
| GAPDH | Forward  Reverse | 5’ – TGC ACC ACC AAC TGC TTA GC – 3’  5’ – GGC ATG GAC TGT GGT CAT GAG – 3’ |
| GFAP | Forward  Reverse | 5’ – AGG TCC ATG TGG AGC TTG AC – 3’  5’ – GCC ATT GCC TCA TAC TGC GT – 3’ |
| ITGA4 | Forward  Reverse | 5’ – AGC CCT AAT GGA GAA CCT TGT – 3’  5’ – CCA GTG GGG AGC TTA TTT TCA T – 3’ |
| KROX20 | Forward  Reverse | 5’ – CCT TTG ACC AGA TGA ACG GAG TG – 3’  5’ – CCT TTG ACC AGA TGA ACG GAG TG – 3’ |
| MPZ | Forward  Reverse | 5’ – CTA TCC TGG CTG TGC TGC TCT T – 3’  5’ – ACT CAC TGG ACC AGA AGG AGC A – 3’ |
| Nanog | Forward  Reverse | 5’ – CAT GAG TGT GGA TCC AGC TGG – 3’  5’ – CCT GAA TAA GCA GAT CCA TGG – 3’ |
| NGFR | Forward  Reverse | 5’ – TGG CCT ACA TAG CCT TCA AGA – 3’  5’ – GAG ATG CCA CTG TCG CTG T – 3’ |
| PMP22 | Forward  Reverse | 5’ – AAT GCT CCT CCT GTT GCT G – 3’  5’ – CAG CAA CAG GAG CAT T – 3’ |
| S100β | Forward  Reverse | 5’ – GAC CCT CAT CAA CGT GTT CCA – 3’  5’ – CCA CAA GCA CCA CAT ACT CCT – 3’ |
| SOX10 | Forward  Reverse | 5’ – CCT CAC AGA TCG CCT ACA CC – 3’  5’ – CAT ATA GGA GAA GGC CGA GTA GA – 3’ |

**Table S2. List of primary antibodies and live cell imaging dyes used in this study**.

| Antibody | Method | Dilution | Company | Catalogue number |
| --- | --- | --- | --- | --- |
| TFAP2 | ICC | 1/500 | Sigma-Aldrich | HPA028850 |
| ATGL | WB | 1/500 | Santa Cruz | SC365278 |
| βIII-Tubulin | ICC | 1/500 | Abcam | ab7751 |
| Di-4-ANEPPDHQ | FACS, SpCI | 1/1000 | Thermo Fisher Scientific | D36802 |
| DGAT1 | WB | 1/2000 | Abcam | ab181180 |
| EGR2/Krox20 | ICC | 1/300 | NovusBio | NBP2-45696 |
| Filipin | ICC | 1/75 | Sigma-Aldrich | F4767-5MG |
| GAP43 | ICC | 1/100 | Abcam | ab75810 |
| GFAP | ICC | 1/500 | Agilent | z033429-2 |
| HNK-1 | ICC | 1/100 | Sigma-Aldrich | C6680 |
| Laurdan | SpCI | 1/500 | Sigma-Aldrich | #40227 |
| LAMP1 (H4A3) | WB | 1/1000 | DSHB | H4A3 |
| LAMP2 (H4B4) | WB | 1/500 | DSHB | SC-18822 |
| LC3B | WB | 1/2000 | Cell Signaling | 3868S |
| LipidSpot™ 488 | ICC | 1/1000 | Biotium | 70065 |
| LysoTracker™555 | ICC | 1/1000 | Thermo Fisher Scientific | L12492 |
| MBP | ICC | 1/500 | Sigma | ab9348 |
| MemBrite® 543/560 | ICC | 1/1000 | Biotium | 30094-T |
| MPZ | ICC | 1/1000 | NovusBio | NB100-1607 |
| mTOR | WB | 1/500 | Cell Signaling | 2983S |
| Nanog | ICC | 1:300 | Thermo Fisher Scientific | PA1-097X |
| NPC1 | WB | 1/2000 | Abcam | ab134113 |
| OCT4 | ICC | 1/100 | Santa Cruz | Sc-9081 |
| NGFR | ICC | 1/500 | Abcam | ab3125 |
| Perilipin-2/ADFP | WB | 1/500 | NovusBio | NB110-40877 |
| PMP22 | IHC | 1/100 | Abcam | ab15506 |
| PMP22 | ICC | 1/100 | Abcam | ab90782 |
| Rab7 | WB | 1/1000 | Cell Signaling | 9367S |
| S100β | ICC | 1/100 | NovusBio | NBP2-59618 |
| Seipin | WB | 1/500 | Abcam | ab106793 |
| SOX10 | ICC | 1/100 | Abcam | ab155279 |
| SOX2 | ICC | 1/500 | Merck Millipore | ab5603 |

FACS = fluorescence activated cell sorting; ICC = immunocytochemistry; IHC = immunohistochemistry;

WB = Western blot; SpCI = spectral confocal imaging.

**Methods**

**Preparation of samples for lipidomics**

For *in vivo* lipidomic analysis, mice were first anesthetized by an i.p. injection with sodium pentobarbital (200 mg/kg: Dolethal). Subsequently, mice were transcardially perfused with PBS, and following this, sciatic nerves were isolated and flash-frozen in ice-cold isopentane. Samples were then stored at -80°C until processed by Lipometrix.

For *in vitro* lipidomic analysis, cells were seeded at 1x10^6^ density per well in 6 well-plates on day 0. On day 1, the medium was changed. On day 3, the cell culture medium was removed, and the cells were gently washed several times with ice-cold PBS on ice. Cells were then collected in a small amount of ice-cold PBS by scraping and centrifuged at 10,000 Relative Centrifugal Force at 4^o^C. PBS was removed, and pellets were stored at -80°C until processed by Lipometrix.

**Lipidomics**

Before the extraction of lipids, cells and mouse sciatic nerves were homogenized in water using an Ultrasonic Tissue Homogenizer UP100H (Hielscher Ultrasonics). For the extraction, an amount of sample containing 10 µg DNA was taken and diluted in 700 μl water. Next, 800 μl of 1 N HCl:CH_3_OH 1:8 (v/v), 900 μl CHCl_3_, 200 μg/ml of the antioxidant 2,6-di-tert-butyl-4-methylphenol (BHT; Sigma-Aldrich) solution were added. For the quantitation of lipids, the samples were spiked with 3 μl of SPLASH® LIPIDOMIX® Mass Spec Standard (#330707, Avanti Polar Lipids), 3 μl of Ceramides and 3 μl of Hexosylceramides Internal Standards (#5040167 and #5040398, AB SCIEX). After vortexing and centrifugation, the lower organic phases were collected, and organic solvents were evaporated using a Savant Speedvac spd111v (Thermo Fisher Scientific) at room temperature. The remaining lipid pellet was stored at -20°C under argon.

Just before mass spectrometry analysis, the precipitates were reconstituted in 100% ethanol. Lipid species were determined via liquid chromatography electrospray ionization tandem mass spectrometry (LC-ESI/MS/MS), using the Nexera X2 UHPLC system (Shimadzu) coupled with hybrid triple quadrupole/linear ion trap mass spectrometer (6500+ QTRAP system; AB SCIEX). Chromatographic separation was performed on an XBridge amide column (150 mm × 4.6 mm, 3.5 μm; Waters) maintained at 35°C. As mobile phase, a 1 mM ammonium acetate in water-acetonitrile 5:95 (v/v) was used, and mobile phase B 1 mM ammonium acetate in water-acetonitrile 50:50 (v/v). The following gradient was applied: 0-6 min: 0% B → 6% B; 6-10 min: 6% B → 25% B; 10-11 min: 25% B → 98% B; 11-13 min: 98% B → 100% B; 13-19 min: 100% B; 19-24 min: 0% B. The initial flow rate was 0.7 ml/min, and it was increased to 1.5 ml/min from 13 min onwards. SM, Cer, HexCer, and Hex2Cer were measured in positive ion mode with a precursor scan of 184.1, and 264.4 for sphingosine-based Cer, HexCer, and Hex2Cer. TG and DG were measured in positive ion mode with a neutral loss scan for one of the fatty acyl moieties. PC, LPC, PE, LPE, PG, PI, and PS were measured in negative ion mode by fatty acyl fragment ions. Lipid quantification was performed by scheduled multiple reactions monitoring (MRM), the transitions being based on the neutral losses or the typical product ions as described above. The instrument parameters were as follows: Curtain Gas (CUR): of 35 psi; Collision Gas: 8 a.u. (medium); IonSpray Voltage (IS): 5500 V (positive ion mode) and −4500 V (negative ion mode); Temperature (TEM): 550°C; Ion Source Gas 1 (GS1): 50 psi; Ion Source Gas 2 (GS2): 60 psi; Declustering Potential (DP): 60 V and −80 V; Entrance Potential (EP): 10 V and −10 V; Collision Cell Exit Potential (CXP): 15 V and −15 V.

The following fatty acyl moieties were taken into account for the lipidomic analysis: 14:0, 14:1, 16:0, 16:1, 16:2, 18:0, 18:1, 18:2, 18:3, 20:0, 20:1, 20:2, 20:3, 20:4, 20:5, 22:0, 22:1, 22:2, 22:4, 22:5 and 22:6 except for TG which considered: 16:0, 16:1, 18:0, 18:1, 18:2, 18:3, 20:3, 20:4, 20:5, 22:2, 22:3, 22:4, 22:5, 22:6. Peak integration was performed with the MultiQuantTM (v 3.0.3, Sciex). Lipid species signals were corrected for isotopic contributions using an in-house-prepared Python script and library Molmass 2019.1.1. Finally, lipid concentrations were computed, using a one-point standard curve approach. According to the guidelines of the Lipidomics Standards Initiative (LSI), a level 2 type quantification was performed in this way. Two matrices of data were used subsequently in the statistical analysis; quantitative with fatty acyl moieties considered as well as sum notations.

**Miniguide notation for lipid structures**

Shorthand notation for lipid structures followed the proposal built upon LIPID MAPS terminology ^1,2^. At the species level, lipid shorthand notations consist of the abbreviation of the lipid class name, followed by the total number of carbons in fatty acyls, colon, and the total number of double bonds. For example, PC 34:1 is phosphatidylcholine with the total number of carbon atoms in both fatty acyls equal to 34 and 1 double bond in the structure. The same rule applies to sphingolipids, with the addition of a semicolon followed by the total number of oxygen atoms (except the amide oxygen). For example, Cer 34:1;O2 refers to a ceramide with a total number of carbon atoms equal to 34, 1 double bond in the structure, and 2 additional oxygen atoms. The molecular species level shows the exact fatty acyl composition; however, sn-1 and sn-2 positions remain unknown, and the “_” separator is used. For example, PC 16:0_18:1, refers to phosphatidylcholine containing FA 16:0 and FA 18:1 attached to the glycerol backbone without knowing their sn-1 or sn-2 positions. In contrast, a “/” separator indicates the exact sn-1 and sn-2 position of constituents (sn-position level). For example, PC 16:0/18:1 refers to phosphatidylcholine with FA 16:0 attached in the sn-1 position and FA 18:1 attached in the sn-2 position, respectively. For sphingolipids, at the molecular species level, the sphingoid base is separated by “/” from the N-linked fatty acid, e.g., Cer 18:1;O2/16:0, indicates a ceramide where a base is a sphingosine containing C18 atoms with one double bond and two hydroxyl groups, and the N-linked fatty acid is FA 16:0.

**Analyzing membrane polarity using flow cytometry and spectral imaging**

Laurdan and Di-4-ANEPPDHQ, which are environmental-sensitive dyes that respond differently depending on membrane polarity ^3^, were used for membrane order experiments. For flow cytometry, iPSC-SCPs were seeded at 1x10^6^ per well in 6 well-plates on day 0. On day 1, the medium was changed. On day 3, cells were stained with Di-4-ANEPPDHQ (Thermo Fisher Scientific; #D36802; 1:1000) for 30 min at 37°C. Following harvesting, cells were washed 3x and resuspended in PBS. Single cells were recorded at excitation wavelengths of fluorescein isothiocyanate (FITC; 495 nm) and phycoerythrin (PE; 566 nm). Values were normalized by subtracting unstained blanks and the excitation generalized polarization (GP_ex_) ^4,5^ was calculated as a measure for membrane order, ranging from -1 (L_d_) to +1 (L_o_).

Equation GP_ex_ via flow cytometry:

$$GP (Di-4-ANEPPDHQ)=\frac{FITC \left( \lambda488 \right)-PE (\lambda555)}{FITC \left( \lambda488 \right)+PE (\lambda555)}$$

To study membrane polarity via spectral imaging, giant plasma membrane vesicles (GPMVs) were generated from CMT1A iPSC-SCPs and isogenic controls as previously described ^6^. Briefly, iPSC-SCPs were cultured for 72 h. Subsequently, the cells were washed with PBS and incubated for 2 h at 37°C with GPMV buffer (10 mM HEPES, 150 mM NaCl and 2 mM CaCl_2_ in MiQ, pH 7.4) supplemented with 25 mM paraformaldehyde (PFA) and 2 mM dithiothreitol (DTT). Next, CMT1A and isogenic GPMVs were collected and labeled by adding either Di-4-ANEPPDHQ (1:1000) or Laurdan (#40227, 6-Dodecanoyl-N,N-dimethyl-2-naphthylamine, Merck, Sigma-Aldrich; 1:500) for 30 min at 37°C in the dark. Labeled GPMVs were divided over different wells in an 8-well microslide chamber (Ibidi) and were allowed to settle for 15 min before imaging. The on-stage specimen temperature was maintained at 23°C.

Spectrally resolved images of the GPMVs were recorded on a Zeiss LSM880 scanhead mounted on the rear port of a Zeiss AxioObserver Z.1 inverted fluorescence microscope equipped with a C-Apochromat 63x/1.2 W Korr M27 water immersion objective lens. Excitation was provided by the 488 nm line of a CW Argon laser used at 0.8% (~17.5 µW recorded at the stage specimen position) for the Di-4-ANEPPDHQ-stained samples. Alternatively, excitation was provided by a tunable MaiTai HPDS DeepSee™ 80 MHz femtosecond pulsed laser set to 810 nm with a requested transmission through the AOM of 3.5% (~19 mW average power on stage) for the Laurdan-stained samples. Excitation and emission light were separated by a MBS488 or a MBS760+ dichroic mirror for the Di-4-ANEPPDHQ and Laurdan samples, respectively. Emission was recorded by the 32-channel Quasar spectral GaAsP detector unit over a spectral range of 410-690 nm (8.9 nm spectral channel width). The pixel dwell time was set to 0.85 µs without additional averaging. An area of 45 µm x 45 µm (Zoom 3) was imaged using 512 x 512 pixels resulting in a pixel size of 88 nm x 88 nm and a total acquisition time of 223 ms. The microscope and acquisition configuration were controlled with ZEN Black 2.6 SP1. Based on the collected spectra, corrected for Quasar spectral response ^7,8^, an emission generalized polarization (GP_em_) value can be calculated as described above. Using Laurdan and Di-4-ANEPPDHQ, a more red-shifted spectrum correlates with less membrane ordering, while a more blue-shifted spectrum correlates with higher membrane order (Supplementary Fig. 8). A custom-written Matlab protocol was used to calculate GP values [based on ^9^]. Briefly, images were processed before corrected spectra were analyzed, which included background subtraction, a Hough transform that selected only fully circular cross-sections displaying proper signal/threshold without “membrane blebbing”, employing openware ImageJ (NIH, Bethesda, MD, USA), and plugins LSM Toolbox, Bio-Formats, as well as academic licensed Matlab (MathWorks®) including Zeiss .czi file reading and the Open Microscopy Environment (OME) script.

Equation GP_em_ via spectral imaging on a confocal system:

$$GP (Laurdan)=\frac{IB(\lambda440) - IR(\lambda490)}{(IB(\lambda440) + IR(\lambda490)}$$

$$GP (Di-4-ANEPPDHQ)=\frac{IB(\lambda560) - IR(\lambda650)}{(IB(\lambda560) + IR(\lambda650)}$$

**Lipid raft dynamic assay and analysis**

CMT1A and isogenic iPSC-SCPs were seeded in 8 well microslide chambers as described above. After 72h, lipid rafts were labeled using a Vybrant™ Alexa Fluor 488 lipid raft labeling kit (V34403, Thermo Fisher Scientific) according to the manufacturer’s instructions. After labeling with cholera toxin b (CTB; lipid raft marker), the medium was refreshed, and cells were allowed to recover for 2 hours. Time series of AF488-labeled lipid rafts were recorded on a Zeiss Elyra PS.1 inverted widefield fluorescence microscope equipped with an automated total internal reflection (TIRF) module and an alpha Plan-Apochromat 100x/1.46 DIC M27 oil immersion objective lens. Excitation was provided by a 488 nm CW laser used at 0.5% (~200 µW recorded from the objective) in objective-based TIRF mode. The TIRF angle was optimized for contrast for each sample (ranging from 65-66°). Excitation and emission light were separated by an MBS 488 dichroic mirror and emission was additionally filtered by a BP495-575 + LP750 emission filter before being recorded by an Andor iXon+ 897 EMCCD camera (512x512 pixels or 16 µm by 16 µm frame size) operating in 16-bit mode with an EM gain of 200 and an exposure time of 33 ms. Additional magnification was provided by a 1.6x optovar lens, resulting in a total magnification of 160x and a pixel size of 100 nm x 100 nm. For each acquisition series, 1000 frames were recorded without delay resulting in a total acquisition time of 33 seconds. The microscope and acquisition configuration were controlled with ZEN Black 2.3 SP1.

Single particle tracking (SPT) was performed by using the Metamorph® software (Molecular Devices) with PALMTracer plugin to quantify and obtain the MSD, diffusion coefficient, and plot highly resolved trajectory maps, as described before ^10^.

**RNA sequencing**

*In vivo*: Tissue was lysed using a Mikro-Dismembrator S (B. Braun Biotech International GmbH, Sartorius group) in TRIzol (Thermo Fisher Scientific). After phenol/chloroform extraction and precipitation, RNA pellet was dissolved in 100 μl buffer RA1 (Machery-Nagel, Duren, Germany). RNA was purified using NucleoSpin RNA XS Kit (Machery-Nagel, Duren, Germany). RNA concentration was measured by using Qubit, RNA-BR Kit (Thermo Fisher Scientific) and quality check was done with a BioAnalyzer RNA Nano Chip (Agilent). cDNA synthesis was performed using NuGEN Ovation RNA-Seq System v2 (7102-A01; NuGEN, San Carlos, CA, USA) followed by purification with the Qiagen MinElute Kit. DNA was sheared to 200 to 400-bp fragments. The DNA was end polished and dA tailed, and adaptors with Bioo barcodes were ligated (Life Technologies). The fragments were amplified (eight cycles) and quantified with a QuBit (Thermo Fisher Scientific). Sequencing was done by Genomescan (Leiden, the Netherlands; https://www.genomescan.nl/) using a Novaseq 6000 PE150 (Illumina). Three to five mice were used per group per timepoint based on previous experiments.

*In vitro*: iPSC-SCPs were seeded at a density of 1x10^6^ per well in 6 well-plates and incubated for 48 h in their culture medium. RNA was extracted from the cells using TRIzol™ reagent. RNA concentration and purity were determined using the Nanodrop ND-1000 (Nanodrop Technologies) and RNA integrity was assessed using a Bioanalyzer 2100 (Agilent). Per sample, an amount of 1 µg of total RNA was used as input. Using the Illumina TruSeq® Stranded mRNA Sample Prep Kit (protocol version: Part # 1000000040498 v00 October 2017) poly-A containing mRNA molecules were purified from the total RNA input using poly-T oligo-attached magnetic beads. In a reverse transcription reaction using random primers, RNA was converted into first-strand cDNA and subsequently converted into double-stranded cDNA in a second-strand cDNA synthesis reaction using DNA Polymerase I and RNAse H. The cDNA fragments were extended with a single 'A' base to the 3' ends of the blunt-ended cDNA fragments after which multiple indexing adapters were ligated introducing different barcodes for each sample. Finally, enrichment PCR was conducted to enrich those DNA fragments that have adapter molecules on both ends and to amplify the amount of DNA in the library. Sequence libraries of each sample were equimolarly pooled and sequenced on Illumina NovaSeq 6000 (S1 flowcell 100 cycles kit v1.5, 100 bp, Single Reads) at the VIB Nucleomics Core ([www.nucleomics.be](http://www.nucleomics.be)).
Analysis of the RNA sequencing data was done by using the <https://unicle.life/> platform analysis.

**Pathway analysis**

RNA-Seq files were processed using the open source BIOWDL RNAseq pipeline v3.0.0 developed at the LUMC. This pipeline performs FASTQ preprocessing (including quality control, quality trimming, and adapter clipping), RNA-Seq alignment and read quantification. FastQC (v0.11.7) was used for checking raw read QC. Adapter clipping was performed using Cutadapt (v2.4) with default settings. RNA-Seq reads were aligned using STAR (v2.7.3a) on the GRCm38 reference genome. The gene read quantification was performed using HTSeq-count (v0.11.2) with setting “–stranded yes”. The gene annotation used for quantification was Ensembl version 99. The raw counts were converted to CPM counts using R package EdgeR (v3.28.1) and from this, fold changes were calculated for every comparison. Data were analyzed through the use of IPA (QIAGEN Inc.)

**Comparing bulk RNA-sequencing data and single-cell atlas of human developing spinal cord**

Bulk RNA-sequencing reads were mapped against the hg38 genome using STAR (v2.7.3a) with default parameters. From the mapped reads a count matrix over genes was generated using featureCounts from the Subread (v1.6.3) package with default parameters. The count matrix of the single-cell atlas of developing human spinal cord along with cell metadata containing cell type annotations ^1^ was downloaded from geo using accession number GSE171890. The single-cell atlas was filtered for annotated cells. To assess transcriptional similarities between the cell types from the single-cell atlas and the bulk RNA-sequencing samples a regression model was fitted with the pseudobulked expression profile from the single-cell atlas as the dependent variable and the bulk RNA-sequencing expression profiles as independent variables, for each cell type in the single cell atlas, using non-negative least squares regression (Scikit-learn, v0.24.1). The regression coefficients from these models were interpreted as a measurement of similarity between the bulk RNA-sequencing samples and the transcriptional profiles of the cell types from the single-cell atlas.

**Myelination assay**

Mouse dorsal root ganglion (DRG) neurons were isolated as described ^11^ and placed in ice-cold neuronal growth medium (Neurobasal medium, B27, and 10 ng/ml Nerve growth factor (NGF)-2.5 s (Merck Millipore), 2 mM L-glutamine, and Pen/Strep (50 U/ml)) with 1/100x RevitaCell supplement. Using sterile fine forceps, DRGs were placed gently one at a time on coverslips previously coated with PLO and laminin and completely dried overnight at 37°C in a 5% CO2 incubator (sterile). Each DRG was encapsulated in drops of 50 µl matrigel 3:1 medium mix. Matrigel was allowed to dry for 1 h at 37°C in 5% CO_2_. Next, the neuronal growth medium was added to the DRGs and incubated overnight. The medium was then supplemented with 10 µM cytosine arabinoside (AraC) to remove contaminating cells and replaced every 24 h for 10 days. iPSC-SCs were then seeded on DRGs and maintained in 50:50 neuronal medium and day 35+ iPSC-Schwann cell medium. After 7 days of co-cultures, the medium was switched to a promyelinating medium which contained 5% FBS, with ascorbic acid added and FSK removed from the medium. The medium was changed 50% every other day and maintained for at least 2 months. It is important to mention that no myelination was observed in the control DRG cultures in which AraC was added to the cultures and iPSC-Schwann cells were omitted (data not shown).

**Sciatic nerve immunohistochemistry analysis**

Sciatic nerves were dissected from WT, C3, and C22 mice and fixed in 10% formalin for one week. Afterward, tissue was embedded in paraffin, and cross-sections of 5 µm thickness were made. Tissue sections were first deparaffinized and rehydrated (from xylene to ethanol 50%, and finally in MilliQ water), and then an antigen retrieval step was performed in a citrate buffer, pH 6.0 (30 min at 96^º^C). Sections were allowed to cool down in phosphate-buffered saline (PBS). Next, the endogenous peroxidase activity was blocked by incubating the sections with 0.3% H_2_O_2_ in methanol for 20 min at room temperature (RT). Afterward, the sciatic nerve sections were blocked for mouse immunoglobulins by using the Dako mouse blocking serum (normal mouse serum in Tris-HCl buffer containing stabilizing protein and 0.015 mol/L sodium azide) for 20 min at RT. Sections were then incubated with the monoclonal mouse anti-human PMP22 antibody (1:100, Abcam, Cambridge, UK, #ab90782) overnight at 4^º^C in a humidified chamber. The following day, sections were washed in PBS and incubated with a goat anti-mouse HRP IgG (1:100, Dako) secondary antibody for 30 min at RT. After several washing steps with PBS, detection of peroxidase activity was achieved with DAB plus (approx. 2-3 min). A Haematoxylin counterstaining was performed to visualize the nuclei. After some final washing steps, the slides were air dried and mounted using Vectashield antifade mounting medium (Vector Laboratories; #H-1000-10).

**High throughput imaging**

The Operetta system (Perkin Elmer) was used for the high throughput image analysis. Cells were seeded on a 96-well plate on day 0 and staining occurred on day 2. For filipin stain, the protocol here reported was followed on the same day of the image acquisition. Cells were washed and incubated for 5 minutes at 37°C with 1X Pre-Staining Solution (MemBrite® Fix 543/560, #30094, Biotium) diluted in DPBS. After a DPBS wash, freshly prepared 1X MemBrite® Fix Dye 543/560 solution (#30094, Biotium) was added for 5 minutes at 37°C. Cells were then washed with DPBS (+/+) and fixed in 4% PFA for 20 min. Cells were then blocked in filtered 5% donkey serum (#D9663, Sigma-Aldrich) for 1 h. Filipin was diluted 1/75 in filtered blocking serum and applied for 2 h at RT in the dark. Cells were then washed once in DPBS (+/+) and subsequently incubated with NucRed® for 20 min followed by DPBS (+/+) wash steps (x3). Cells were imaged immediately after staining using the Operetta system and image analysis was performed via the provided Harmony software. Cells were segmented with the help of the digital phase contrast images and PM filipin staining intensity was analyzed. For LD and lysosome accumulation, DAPI, LipidSpot, and LysoTracker, were incubated with cells 30 min prior to imaging. Just before imaging started, the medium was replaced with SCPDM made with DMEM/F12 phenol red-free medium (Thermo Fisher Scientific; #21041025) with oleic acid (OA) (Sigma-Aldrich) (concentration ranged from 10-500 µM for different experiments). Half the normal concentration of LipidSpot and LysoTracker (Supplementary Table 2) were added to the medium during live cell imaging. Analysis of high throughput imaging was done using the Harmony software, through which LDs and lysosomes number and size could be evaluated over time.

For the treatment of iPSC-SCPs with a cAMP activator, forskolin (FSK) was added to the medium 1 h before analyzing the cells on the Operetta system. For the treatment of the iPSC-SCPs with a progesterone receptor antagonist, PF-02413873 (Sigma-Aldrich; #PZ0367-5MG) was added to the cell culture medium. After either 3 h or overnight treatment, iPSC-SCPs were then analyzed by filipin staining.

**Transmission electron microscopy on iPSC-SCPs**

CMT1A and isogenic iPSC-SCPs were cultured for 48-72 h without any medium change until cells reached 70% confluency and fixed using 5% glutaraldehyde (1 h, RT) followed by 2.5% glutaraldehyde (overnight, 4°C) in 0.1 M sodium-cacodylate buffer. Fixed iPSC-SCPs were scraped in the presence of 0.1 M sodium-cacodylate buffer, centrifuged (200xg, 10 min) and cell pellets were resuspended and incubated in 1.5% agarose for 30 min at 4°C. The embedded cell pellets were post-fixed in 1% osmium tetroxide (2 h, RT in the dark), rinsed with dH_2_O, and dehydrated in a graded ethanol series (30-100%). Samples were stained en bloc overnight with 4% uranyl acetate in the 70% ethanol step at 4°C. Finally, after propylene oxide treatment (2 changes 15 min each), pellets were infiltrated with and embedded in 100% epoxy resin for two days (60°C). Ultrathin sections (50 nm) were post-stained with 3% uranyl acetate in water (10 min), and in Reynold’s lead citrate (2 min). Micrographs were taken with a JEOL JEM1400 (JEOL, Japan) at 80 KV.

Quantification of late endosome-lysosomes (LELs): Ultrathin sections of iPSC-SCPs were analyzed with magnifications of between 5000-12000x using transmission electron microscopy. If LELs were extensively touching or fusing with other LELs or organelles, perimeter size was not estimated. Images of a cell represent one image plane within a cell and are not representative of the total LELs per whole cell.

**Supplementary Figures**


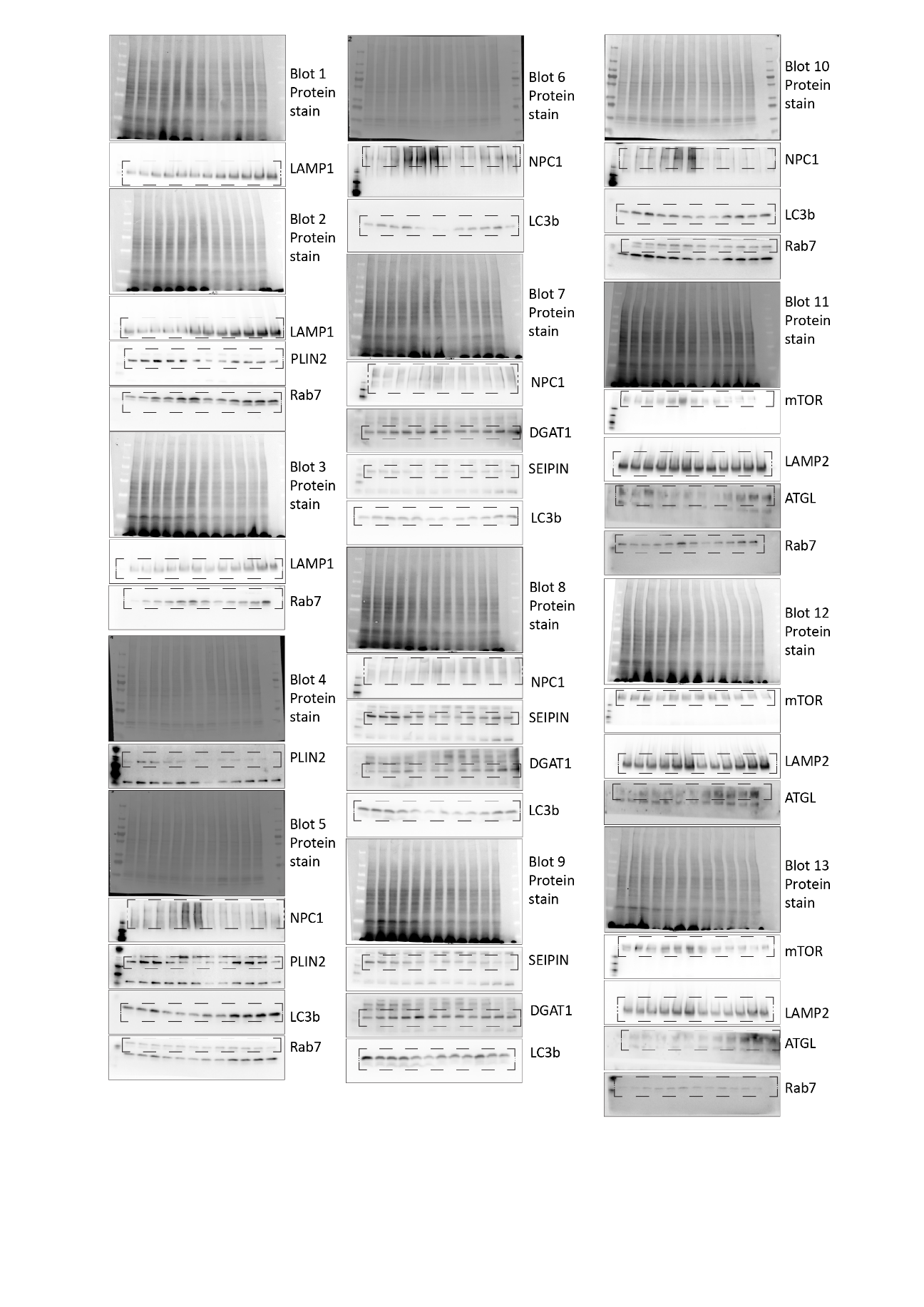


**Supplementary Figure 1. Overview of Western blots used for quantifications in Figure 6 of the main text.** 5 experiments with markers stained at least 3 times for quantifications. After protein stain, markers stained for each blot are highlighted. Blots are not ordered in a sequential manner.


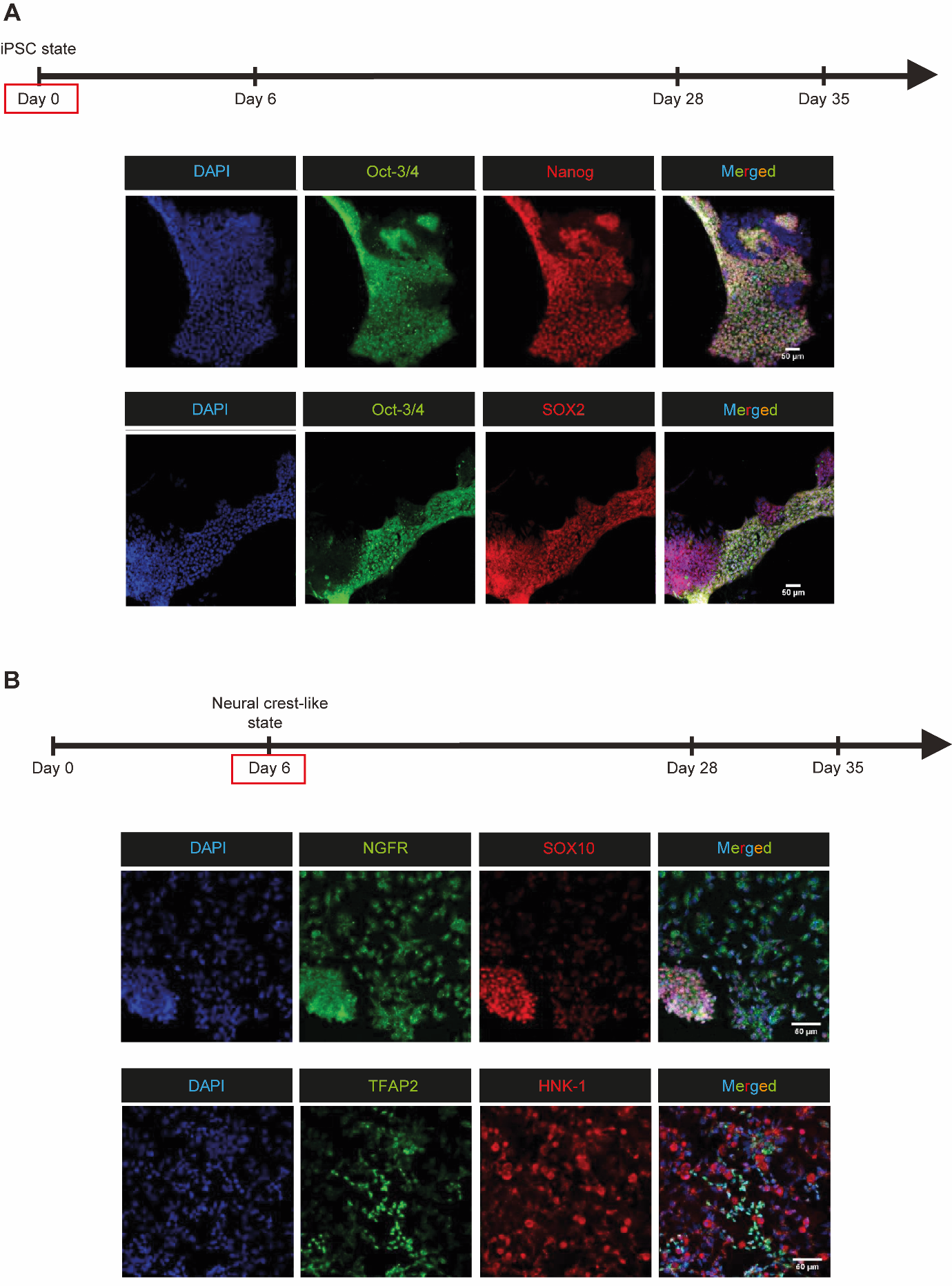


**Supplementary Figure 2. Expression of pluripotency and neural crest markers from days 0-6 of the iPSC-Schwann cell differentiation protocol.** The control cell line from Sigma was used for the initial characterization of the iPSC-Schwann cell differentiation protocol. Their differentiation in the early stages of the protocol included validation of the expression of the **A)** pluripotency markers Oct-3/4, Nanog, and SOX2, and of the **B)** early neural crest markers, NGFR, TFAP2A, HNK-1, and SOX10 via immunocytochemistry. Nuclei (in blue) are visualized using DAPI. Scale bar is 50 µm for all images.

**
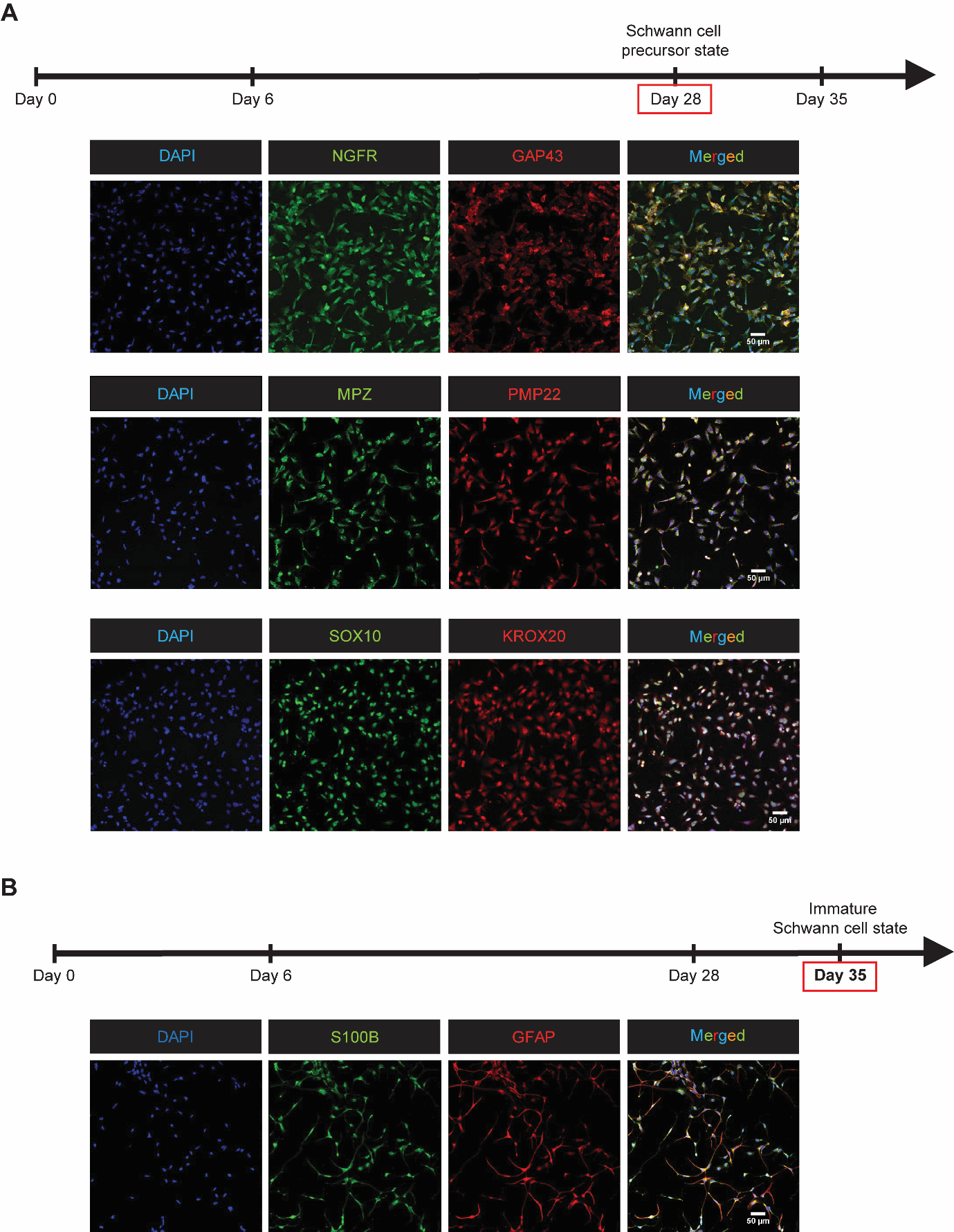
**

**Supplementary** **Figure 3. Expression of precursor and immature Schwann cell markers from days 28-35 of the iPSC-Schwann cell differentiation protocol.** The control cell line from Sigma was used for the initial characterization of the iPSC-Schwann cell differentiation protocol. **A)** Their differentiation at the later stages of the protocol included expression analysis of Schwann cell precursor (SCP) markers such as NGFR, GAP43, MPZ (P0), PMP22, SOX10, and KROX20/EGRF2 on the protein level via immunofluorescence. **B)** At the final step, differentiated iPSCs are positive for the Schwann cell markers S100β and GFAP, and they have a bi-tripolar morphology. Nuclei (in blue) are visualized using DAPI. Scale bar is 50 µm in each image.


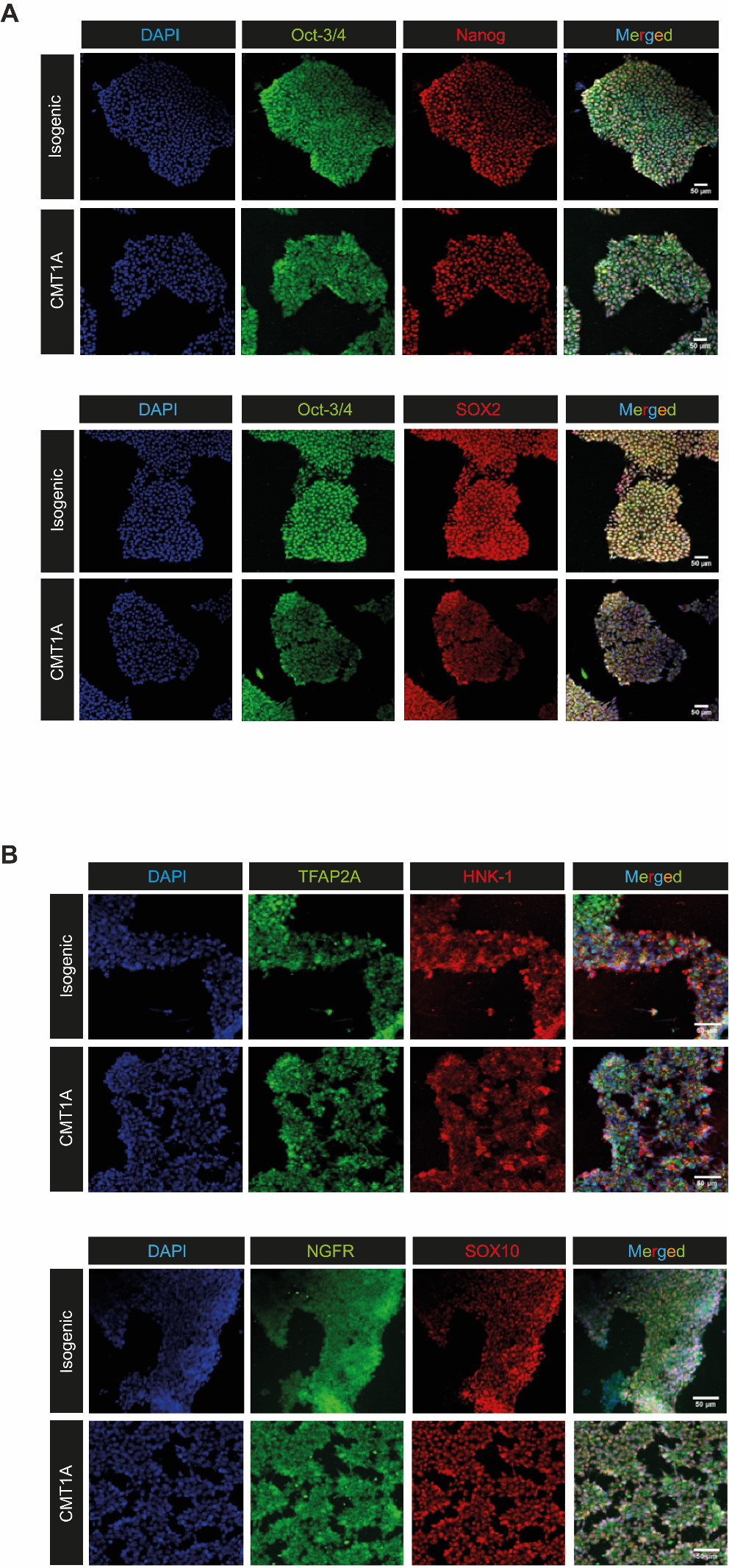


**Supplementary** **Figure 4. Expression of pluripotency markers of the CMT1A and isogenic iPSC line at the start of the differentiation protocol (iPSC-state) and after 6 days (neural crest level).** **A)** Both the CMT1A and its isogenic control line are positive for several pluripotency markers, such as Nanog, Oct-3/4, and SOX2 at an iPSC level at the start of the differentiation. **B)** On day 6 of the protocol, the neural crest markers TFAP2A, HNK-1, NGFR, and SOX10 start to become expressed in both CMT1A and isogenic iPSCs. DAPI (in blue) was used as a nuclear counterstain. The scale bar is 50 µm in each image.


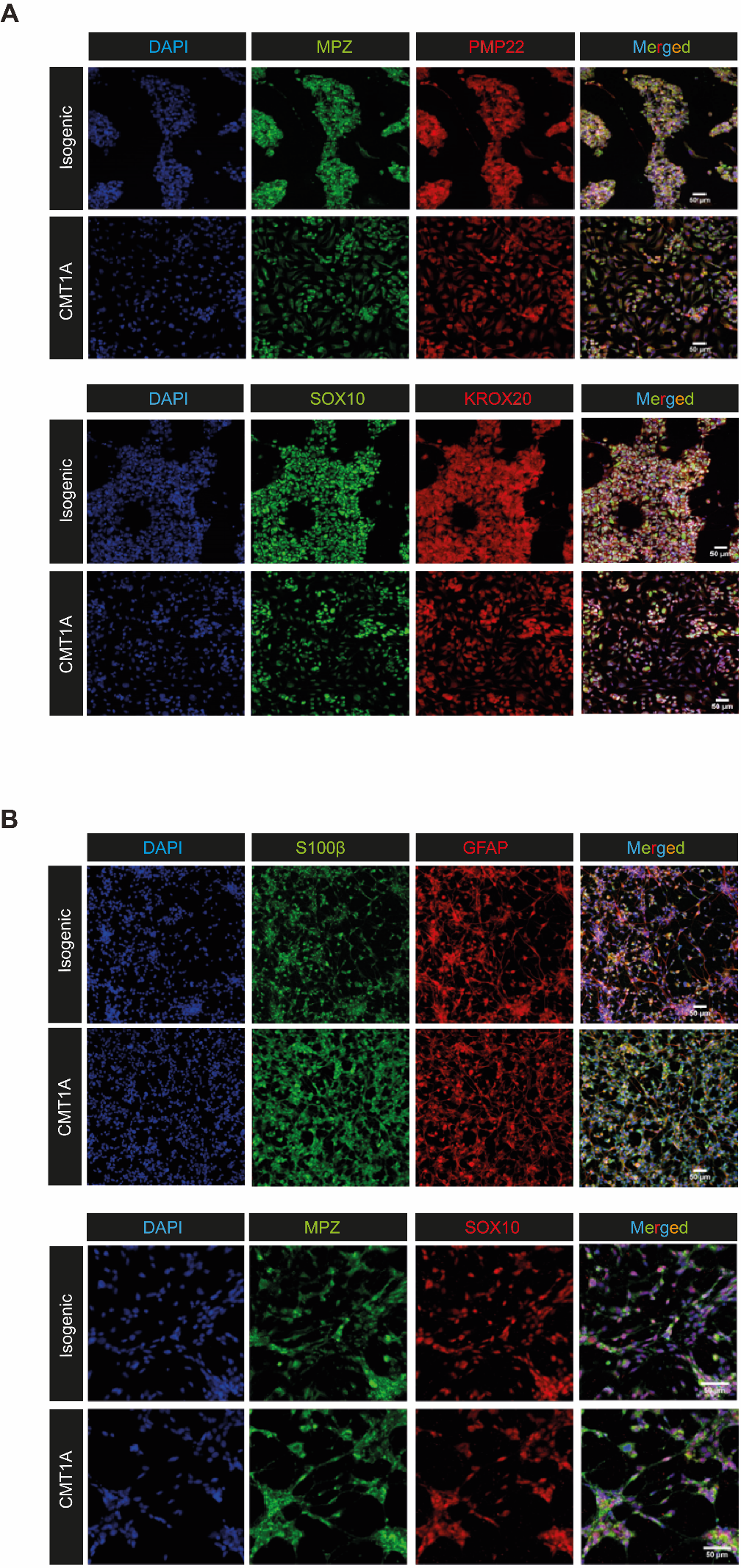


**Supplementary** **Figure 5. Expression of precursor and immature Schwann cell markers from day 28-35 of the iPSC-Schwann cell differentiation protocol for the CMT1A and its isogenic line. A)** Both the CMT1A and its isogenic control line stained positive for MPZ (P0), PMP22, KROX20/EGRF2, and SOX10 on day 28 of the protocol. **B)** In addition, at day 35 of the differentiation protocol both the CMT1A and isogenic iPSC-SCPs stained positive for S100β, GFAP, MPZ (P0), and SOX10. DAPI (in blue) was used as a nuclear counterstain. The scale bar is 50 µm in each image.


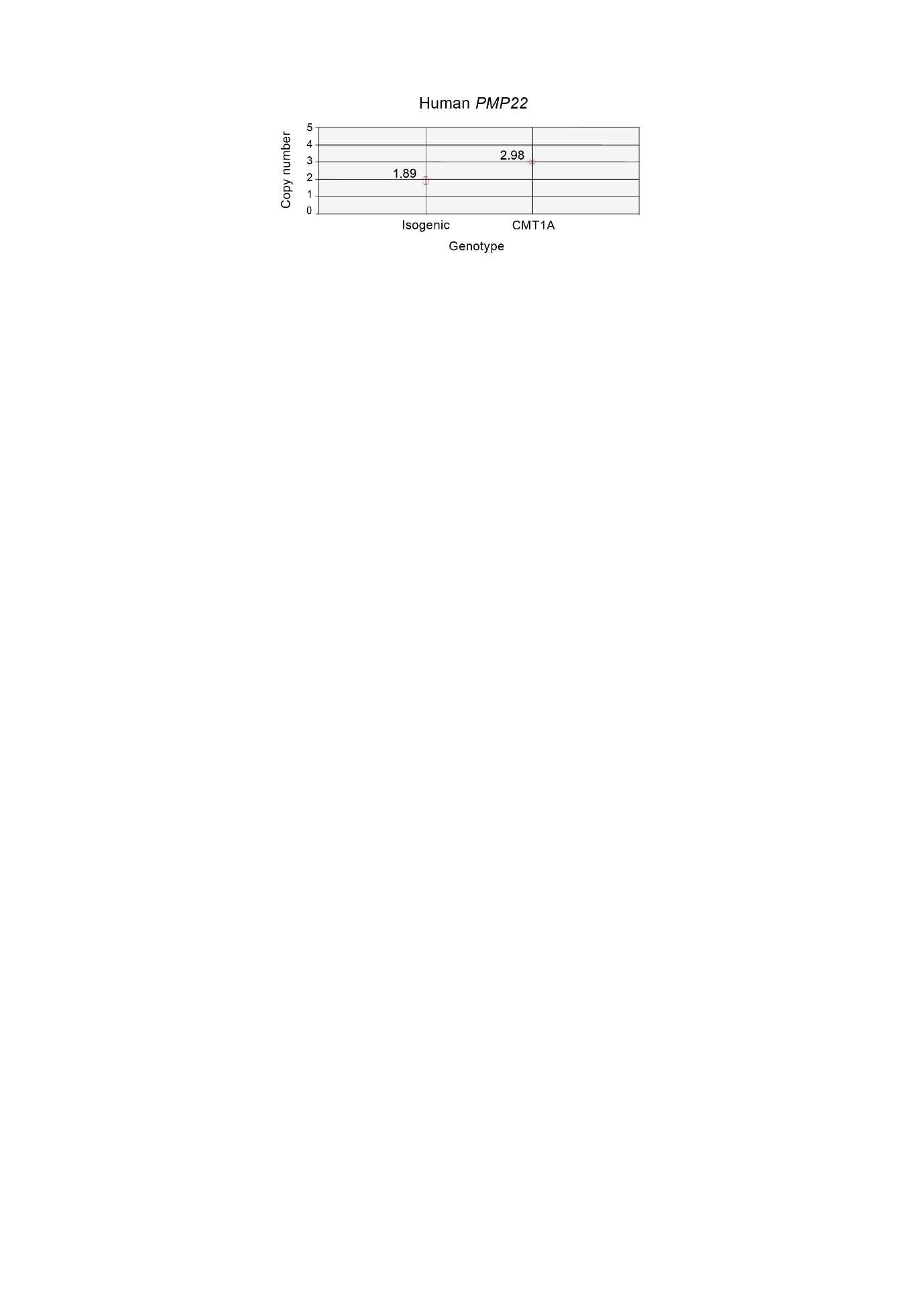


**Supplementary Figure 6. ddPCR analysis of isogenic and CMT1A iPSC-SCPs at 28 day of the Schwann cell differentiation protocol.** Isogenic iPSC-SCPs express 1.89 copies of the human PMP22 and CMT1A iPSC-SCPs 2.98 copies, exactly one copy extra compared to the isogenic line.


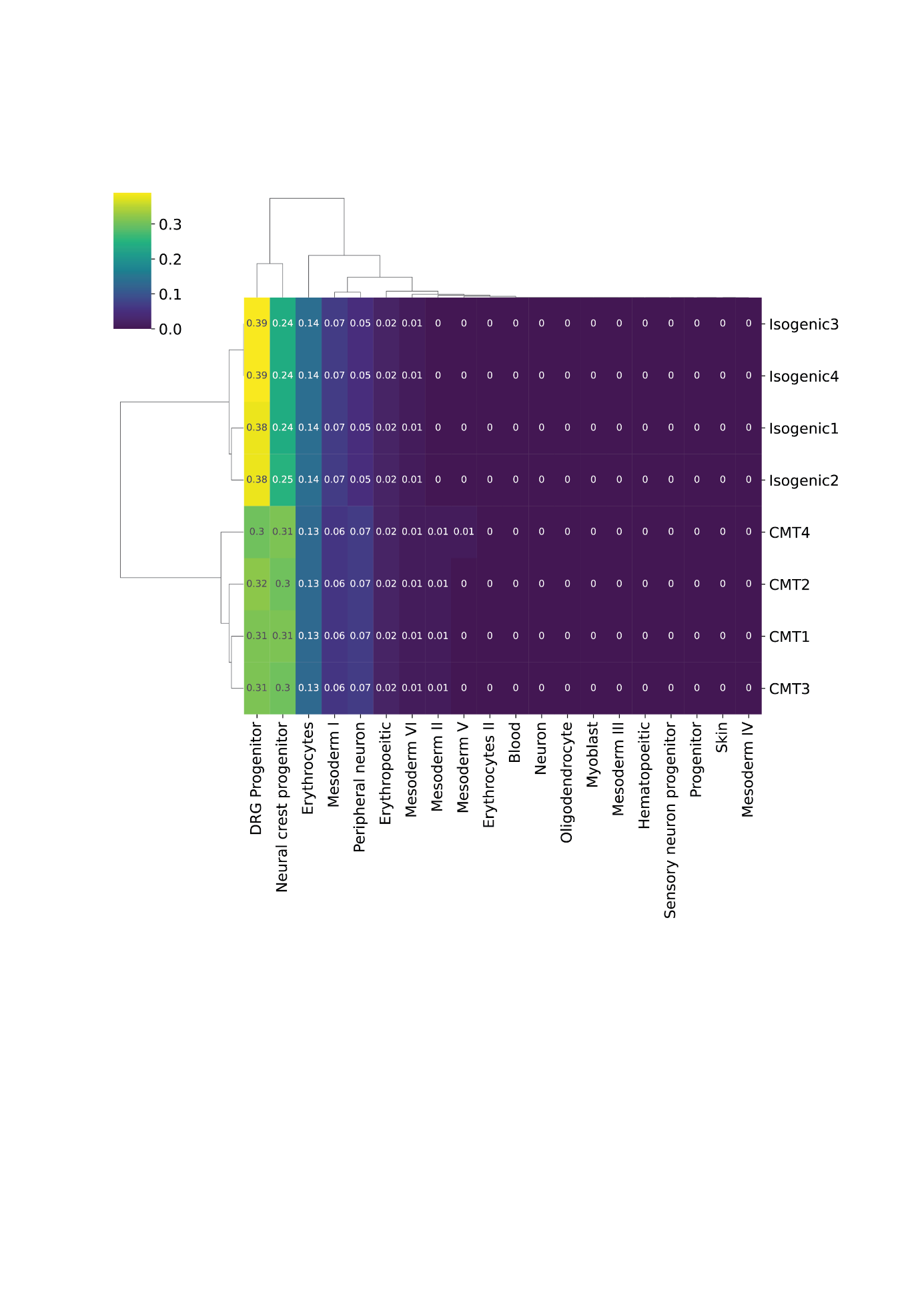


**Supplementary Figure 7. Bulk RNA-sequencing data maps to neural crest and DRG progenitor cells from a single cell atlas of a human developing spinal cord.** DRG progenitors, which are defined as predominantly SOX10 positive, and neural crest progenitors, which are predominantly SOX10 and SOX2 positive cells, were the major cell types when mapped to the single cell atlas of the human developing spinal cord ^11^. The term “Schwann cell precursor” was not used in the annotation of cell clusters in the study by Rayon *et al.* ^11^, therefore it could not be used in the current cell cluster annotation in this figure.


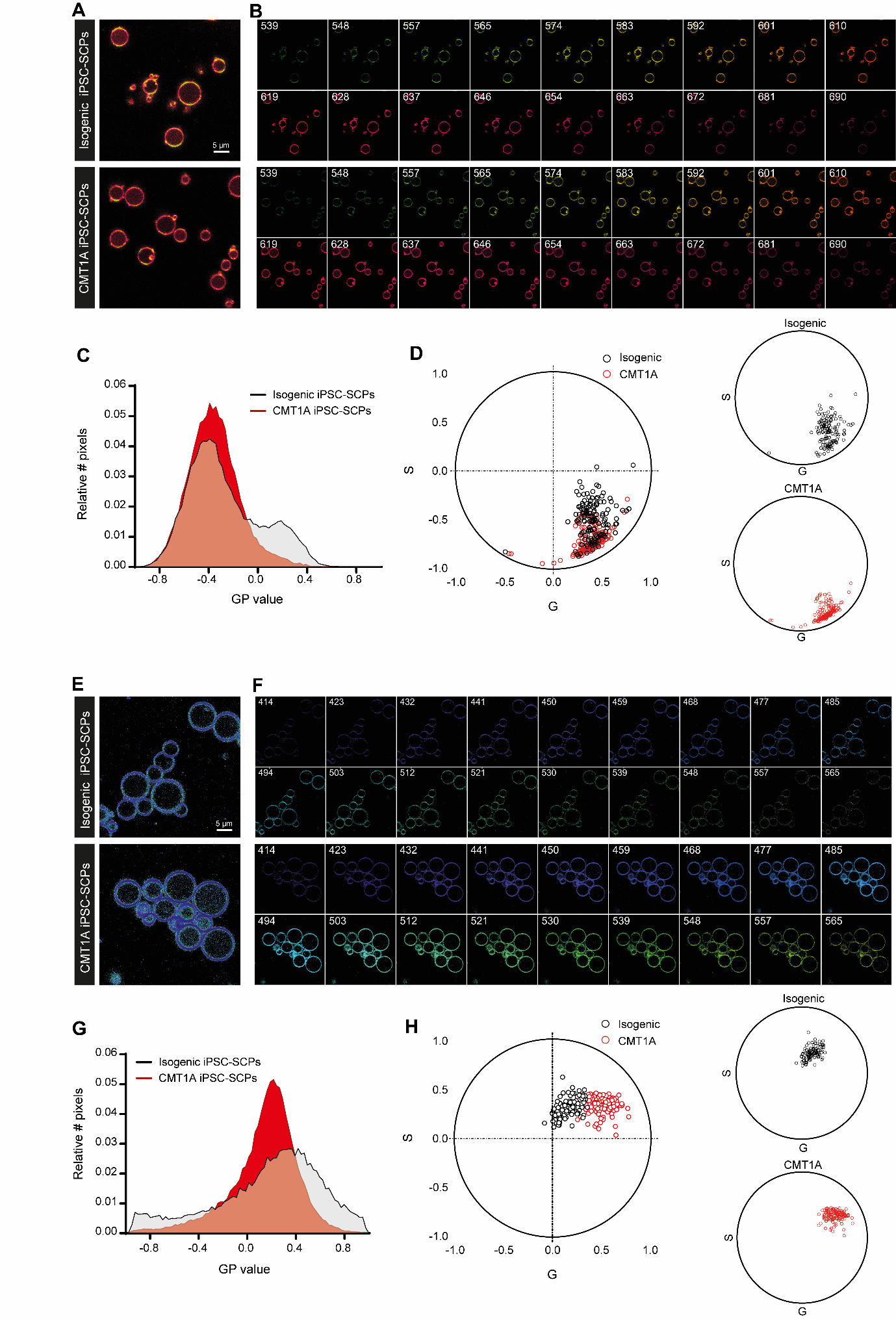


**Supplementary Figure 8.** **Spectral imaging with Di-4-ANEPPDHQ and Laurdan indicates decreased membrane order in the plasma membrane of CMT1A iPSC-SCPs.** GPMVs were generated, and spectral (lambda, λ) confocal imaging was performed at different wavelengths with either Di-4-ANEPPDHQ or Laurdan as described in the methods. **A, B)** Photomicrographs of Di-4-ANEPPDHQ stained GPMVs from isogenic vs CMT1A iPSC-SCPs, including images with split wavelengths that are used to calculate the GP_em_ = (I_B(λ560)_ – I_R(λ650)_) / (I_B(λ560)_ + I_R(λ650)_), ranging from -1 (liquid disordered, Ld) to +1 (liquid ordered, Lo). CMT1A: n = 164 and isogenic: n = 142 GPMVs analyzed, three independent experiments, data of one representative experiment are shown. **C)** Histogram indicating the distribution and number of pixels per GP value for GPMVs from CMT1A (in red) and isogenic (in black) iPSC-SCPs labelled with Di-4-ANEPPDHQ. Histogram indicating the distribution and number of pixels per GP value for Di-4-ANEPPDHQ GPMVs. **D)** Spectral phasor plots also indicate significant differences in data distribution between isogenic and CMT1A GPMVs (multivariate ANOVA analysis, *P* < 0.0001, Eta Squared = 0.35). **E, F)** Photomicrographs of Laurdan stained GPMVs from isogenic vs CMT1A iPSC-SCPs, including images with split wavelengths that are used to calculate the GP = (I_B(λ440)_ – I_R(λ 490)_) / (I_B(λ 440)_ + I_R(λ 490)_). CMT1A: n = 151 and isogenic: n = 112 GPMVs analyzed, two independent experiments, data of one representative experiment are shown. **G)** Histogram indicating the distribution and number of pixels per GP value for GPMVs from CMT1A (in red) and isogenic (in black) iPSC-SCPs labelled with Laurdan. **H)** Spectral phasor plots also indicate significant differences in data distribution between isogenic and CMT1A GPMVs (multivariate ANOVA analysis, *P* < 0.0001, Eta Squared = 0.73).

**References**

1. Liebisch G, Vizcaíno JA, Köfeler H, et al. Shorthand notation for lipid structures derived from mass spectrometry. *J Lipid Res*. 2013;54(6):1523-1530. doi:10.1194/jlr.M033506

2. Liebisch G, Fahy E, Aoki J, et al. Update on LIPID MAPS classification, nomenclature, and shorthand notation for MS-derived lipid structures. *J Lipid Res*. 2020;61(12):1539-1555. doi:10.1194/jlr.S120001025

3. Parasassi T, Gratton E. *Membrane Lipid Domains and Dynamics as Detected by Laurdan Fluorescence*. Vol 5.; 1995.

4. Sezgin E, Levental I, Mayor S, Eggeling C. The mystery of membrane organization: Composition, regulation and roles of lipid rafts. *Nat Rev Mol Cell Biol*. 2017;18(6):361-374. doi:10.1038/nrm.2017.16

5. Hoffmann K, Nirmalananthan-Budau N, Resch-Genger U. Fluorescence calibration standards made from broadband emitters encapsulated in polymer beads for fluorescence microscopy and flow cytometry. *Anal Bioanal Chem*. 2020;412(24):6499-6507. doi:10.1007/s00216-020-02664-y

6. Velapoldi RA, Tønnesen HH. *Corrected Emission Spectra and Quantum Yields for a Series of Fluorescent Compounds in the Visible Spectral Region*. Vol 14.; 2004.

7. Brewer J, de la Serna JB, Wagner K, Bagatolli LA. Multiphoton excitation fluorescence microscopy in planar membrane systems. *Biochim Biophys Acta Biomembr*. 2010;1798(7):1301-1308. doi:10.1016/j.bbamem.2010.02.024

8. Slenders E, Seneca S, Pramanik SK, et al. Dynamics of the phospholipid shell of microbubbles: A fluorescence photoselection and spectral phasor approach. *Chemical Communications*. 2018;54(38):4854-4857. doi:10.1039/c8cc01012a

9. Bademosi AT, Lauwers E, Padmanabhan P, et al. In vivo single-molecule imaging of syntaxin1A reveals polyphosphoinositide- and activity-dependent trapping in presynaptic nanoclusters. *Nat Commun*. 2017;8. doi:10.1038/ncomms13660

10. Kechkar A, Nair D, Heilemann M, Choquet D, Sibarita JB. Real-Time Analysis and Visualization for Single-Molecule Based Super-Resolution Microscopy. *PLoS One*. 2013;8(4). doi:10.1371/journal.pone.0062918

11. Richner M, Jager SB, Siupka P, Vaegter CB. Hydraulic extrusion of the spinal cord and isolation of dorsal root ganglia in rodents. *Journal of Visualized Experiments*. 2017;2017(119). doi:10.3791/55226

12. Rayon T, Maizels RJ, Barrington C, Briscoe J. Single-cell transcriptome profiling of the human developing spinal cord reveals a conserved genetic programme with human-specific features. *Development (Cambridge)*. 2021;148(15). doi:10.1242/DEV.199711
